# Optimal two-point loading strategy for accurate leg press force–velocity assessment

**DOI:** 10.1371/journal.pone.0356566

**Published:** 2026-08-25

**Authors:** Takuya Nishioka, Shota Yamaguchi, Ayumi Yoshikawa, Naoki Ikeda, Daiki Hajima, Naoko Yabuno, Yasuhiro Kunita, Yuta Hashimoto, Anna Aoki, Miina Takahashi, Kazunori Nosaka, Takayuki Inami

**Affiliations:** 1 Institute of Physical Education, Keio University, Yokohama, Kanagawa, Japan; 2 Graduate School of Health Management (Sport and Health Sciences), Keio University, Fujisawa, Kanagawa, Japan; 3 School of Medical and Health Sciences, Edith Cowan University, Joondalup, Western Australia, Australia; UC: University of Calgary, CANADA

## Abstract

Accurate assessment of an individual force–velocity (F–V) profile is essential for prescribing targeted resistance training; however, F–V outcomes depend strongly on the testing method used. This study examined the concurrent validity of the F–V relationship in a horizontal leg press exercise by comparing two-point methods, which use pairs of different loads relative to body weight, with a multiple-point method using a wider range of loads. Sixteen men (mean ± SD, age: 20.0 ± 1.4 years, height: 172.4 ± 6.3 cm, body mass: 69.2 ± 7.6 kg) performed maximal-effort horizontal dynamic leg press actions on a device equipped with pneumatic artificial muscles (ddrobotec^®^, Switzerland) against eleven external loads (100%–300% of body weight [BW] in 20% increments). The F–V relationship was obtained using 10 two-point methods that paired a 100% BW load with each of the additional loads (120%–300% BW) and also from all points. The F–V relationship parameters were compared between the two-point methods and the multiple-point method, and their correlations were analyzed. The results showed that the F–V relationship obtained from the four two-point methods with wider load differences (100% and 240%, 100% and 260%, 100% and 280%, and 100% and 300% BW) was nearly perfectly correlated with that by the multiple-point method (*r* = 0.906 to 0.993). The concurrent validity of the F–V relationship parameters obtained from the two-point methods tended to decrease when using loads that were closer together (e.g., 100% and 120% BW) than loads with a larger difference (e.g., 100% and 240% BW). These findings indicate that, on a horizontal leg press with pneumatic artificial muscles, a two-point method based on sufficiently distinct loads can provide an accurate estimate of the F–V relationship and may be a practical alternative to comprehensive multi-load testing.

## Introduction

Leg extensions are performed during various athletic activities, such as vertical jumps and sprint running [1,2], and horizontal dynamic leg press exercises are widely used to train and evaluate leg extension performance [1,3–8]. Our research team recently reported high reliability in measuring force, velocity, and power during the horizontal dynamic leg press exercise using a device equipped with pneumatic artificial muscles (ddrobotec^®^ System ELITE mk4, Dynamic Devices, Zurich, Switzerland), with some variables showing higher reliability than those measured in vertical jumps [1]. Furthermore, unlike the vertical jump, the horizontal dynamic leg press avoids the landing impact, making it a potentially useful option for researchers and practitioners seeking to measure performance with a lower injury risk [1,4]. Taken together, the high measurement reliability and the absence of landing impact can make the horizontal dynamic leg press a particularly suitable model for evaluating F–V profiling methodology, in which a wide range of loads must be repeatedly tested.

Explosive athletic performance—jumping and sprinting—relies heavily on maximum power production during leg extension movements [9–11]. Since power is the product of force and velocity, it is lower when force and/or velocity are reduced [8,12]. Although force-velocity-power measurements are often conducted using a single load (e.g., body weight [BW]), this approach does not allow evaluation of an individual’s maximum force, velocity, and power production capability [13]. Therefore, investigators have used methods to determine the force–velocity (F–V) relationship for a given task through measurements under multiple load conditions [14]. Parameters such as the theoretical maximum force (F0), velocity (V0), power (Pmax), and slope of the linear F–V relationship (SFv) obtained through F–V profiling have been used to identify individual weaknesses and develop more effective training programs [8,14].

The F–V profile for leg press exercises is typically based on 6–11 data points with different loads [15,16]. However, including more data points increases the time required for the assessments, which burdens both investigators and athletes. Considering the high linearity of the F–V relationship reported for leg press exercises [15–17], the number of required data points could be reduced to two [18,19]. This two-point method can simplify the F–V profiling, providing quick and less fatigable measurements [20]. Nevertheless, the usefulness of the two-point method for F–V profiling in leg press exercises has not yet been validated. Thus, it is necessary to investigate the validity of various two-point methods in leg press exercises.

The purpose of this study was to compare the concurrent validity of the F–V relationship parameters in leg press exercises obtained from two-point methods using two different loads relative to BW against the multiple-point method, in which many loads were used. Based on previous studies [20,21], we hypothesized that the concurrent validity of the F–V relationship parameters obtained from the two-point method with different loads relative to the multiple-point method would be high, whereas the validity would progressively decrease when using closer loads.

## Materials and methods

### Experimental design

A repeated-measures design was used to examine the concurrent validity of F–V relationship parameters obtained from various two-point methods with loads differing in intensity relative to body weight in comparison to the multiple-point method, in which many loads were used to determine the F–V relationship during a leg press exercise. A familiarization session was conducted on the first day, and the main performance measurement was obtained on the second day, which was set 48–144 hours later depending on the availability and convenience of the participants and investigators, in accordance with previous studies [6,22].

### Participants

This study was conducted in Japan from December 16, 2022, to March 31, 2024. The sample size was calculated using the G*Power software (version 3.1.9.6, Düsseldorf, Germany) to determine the number of participants required to detect a significant correlation between the F–V relationship parameters obtained from the two-point and multiple-point methods, which was the primary statistical analysis addressing the main aim of this study. Based on an expected correlation coefficient of 0.7 [20], a significance level of 0.05, and a statistical power of 0.95, the required sample size was calculated to be 16 participants. Accordingly, 16 men were recruited, with the following mean ± SD (range): age 20.0 ± 1.4 (18–23) years, height 172.4 ± 6.3 (163–186) cm, and body mass 69.2 ± 7.6 (54.3–86.0) kg, and resistance-training experience, including lower-body exercises, 2.5 ± 1.6 (0.5–5.0) years. Only men were recruited for the present study because the participants were required to perform the leg press exercise with heavier loads, and men generally have greater lower-limb strength than women. All participants self-reported their sex as male. They were free from musculoskeletal pain or injury that could affect testing and were instructed to refrain from strenuous exercise during the 24 hours preceding each experimental session. After the study purpose, procedures, risks, and benefits were explained, written informed consent was obtained before participation. This study was conducted in accordance with the principles of the Declaration of Helsinki and was approved by the ethics committee of our university (approval number: 22-012; approved on December 16, 2022).

### Testing procedures

Measurements during the leg press were taken using a horizontal dynamic leg press machine (ddrobotec^®^ System ELITE mk4, Dynamic Devices, Zurich, Switzerland) [1]. The seat position was individually adjusted to achieve a knee flexion angle of approximately 90° with both feet placed symmetrically on the footplates. Participants began with two familiarization trials at the lightest load, corresponding to 100% of their BW. Thereafter, the load was gradually increased in fixed steps of 20% BW from 100% to 300% BW, based on a previous study [1]. Participants performed two trials for each load, with a rest period between them. The rest periods progressively increased with increasing load; for the first three load levels, the rest intervals ranged from 10 to 20 seconds, and for the remaining loads, from 20 to 40 seconds [1,5,6]. Participants were instructed to extend both legs forcefully and rapidly during each trial; however, no verbal encouragement or feedback was provided. Pneumatic semi-isotonic resistance prevented maximal effort from producing ballistic action, and the entire push-off was performed with maximal intentional velocity [1,5,6]. The leg press was performed as a concentric-only action without preceding countermovement, as the pedals rested in their consistent starting position before each repetition. A horizontal dynamic leg-press device passively guided the eccentric phase, which was not recorded.

### Data analyses

The ddrobotec^®^ System ELITE recorded the force and footplate displacement data (i.e., arc length) at a sampling frequency of 200 Hz using the integrated pressure sensors and position transducers of the horizontal dynamic leg press device [1]. Given the upper limit of this device’s sampling frequency (200 Hz), no digital filters were applied to these data to avoid over-smoothing. The displacement of the footplates, derived from system-specific pre-calibration data, was used to estimate knee joint angles for each participant. Before the first measurement session, each participant was calibrated once using the manufacturer’s two-point procedure: with the lower back firmly against the backrest, the pedal positions corresponding to 5° and 90° of knee flexion were recorded. The device’s internal algorithm subsequently mapped pedal angle to anatomical knee angle from these two reference points across the full range of motion. The instant when the estimated knee joint angles began to move in the direction of extension for both legs was defined as the initial range of motion, and the instant when the estimated knee joint angle decreased to <10° was defined as the end of motion. Force values were computed as the sum of the forces measured from both pedals and normalized to each participant’s body mass. The velocity values were calculated by dividing the arc length of the footplate over the range of motion by the completion time. Velocity values were computed as the average of the velocities measured from both pedals. For force and velocity values, the average of the two trials for each load was used for the F–V profiling described below.

**Fig 1:**
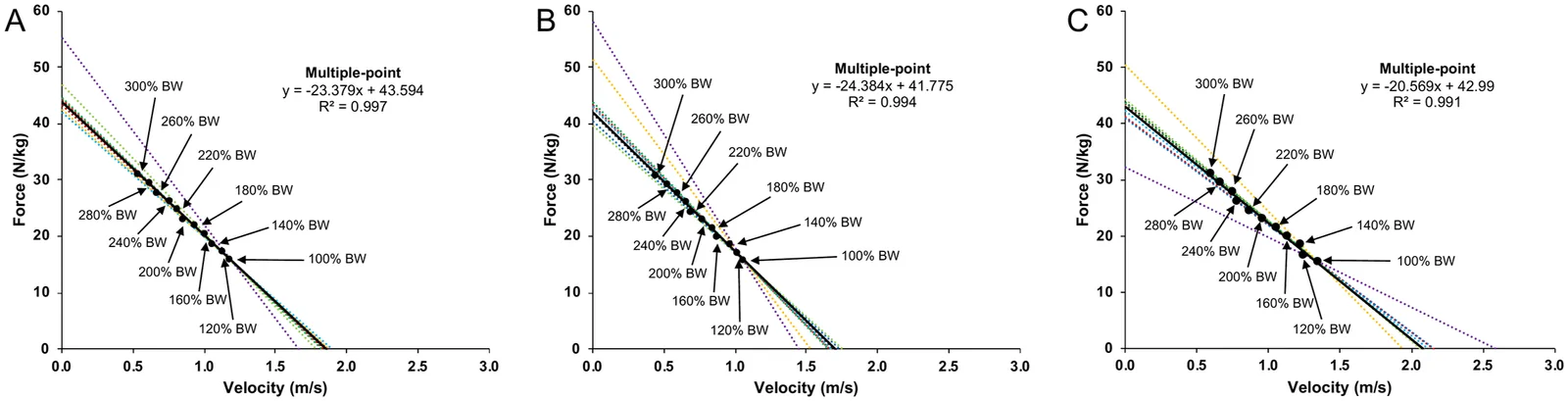
Typical leg press force–velocity (F–V) relationships of three individuals (A, B, C) showing different F–V profiles. The black solid line in each panel represents the regression line from the multiple-point method using all loads, with its formula inserted. The dotted lines represent 10 two-point methods, each combining 100% of body weight (BW) with a heavier load ranging from 120% to 300% of BW in 20% increments. Two-point method load pairs with 100% BW: 300% (black), 280% (gray), 260% (dark red), 240% (red), 220% (blue), 200% (light blue), 180% (green), 160% (light green), 140% (orange), 120% (purple).

Mean force and velocity data obtained under 11 different loads (100%–300% BW) were modeled using a least-squares linear regression to determine the F–V relationship parameters through the multiple-point method: F(V) = F0−aV, where F0 denotes the theoretical maximum force (force-intercept), and V0 denotes the theoretical maximum velocity (velocity-intercept); the slope of the linear F–V relationship was calculated as SFv = −F0/V0 [8,14,20]. As a consequence of the F–V relationship being highly linear, the maximum power (Pmax) was computed as Pmax = F0·V0/4. The same F–V relationship parameters were calculated using 10 two-point methods that paired a 100% BW load with each of the additional loads (120%–300% BW) (Fig 1). These load selections were based on a previous study [20] that used the two-point method for vertical jumps and consistently included a 100% BW load.

### Statistical analyses

Statistical analyses were performed using the Statistical Package for the Social Sciences (SPSS software, version 29; IBM Corp., Armonk, NY, USA) and Microsoft Excel [23]. The significance level was set at *P* < 0.05. Normality was confirmed using the Shapiro–Wilk test. The intraclass correlation coefficient (ICC; two-way mixed effects, absolute agreement, single measurement) [24] was calculated to analyze relative reliability, and the coefficient of variation (CV) and standard error of measurement (SEM) [25] were calculated to analyze absolute reliability. ICC interpretation thresholds followed existing reporting guidelines: < 0.50 = poor, 0.50–0.75 = moderate, 0.75–0.90 = good, and >0.90 = excellent [24]. The CV was interpreted using the following scale: > 15% = poor; 10%–15% = moderate; 5%–10% = good; < 5% = excellent. Paired-sample *t*-tests and effect sizes (Cohen’s *d*) were used to compare *t*he magnitudes of the F–V relationship parameters between the two- and multiple-point methods. The magnitude of Cohen’s *d* was interpreted as follows: trivial (<0.20), small (0.20–0.60), moderate (0.60–1.20), large (1.20–2.00), very large (2.00–4.00), and extremely large (>4.00) [26]. The mean percent bias was interpreted a priori as poor (>10%), moderate (5–10%), or good (<5%), based on percent bias thresholds reported in previous research [27]. Pearson’s *r* was used to assess the relationship between the two- and multiple-point methods. The strength of the correlations was interpreted as follows: negligible (<0.10), weak (0.10–0.30), moderate (0.30–0.50), strong (0.50–0.70), very strong (0.70–0.90), or nearly perfect (>0.90) [26]. The 95% confidence intervals (CIs) for the ICC, CV, bias, and Pearson’s *r* were calculated. Bland–Altman plots were generated, providing a comprehensive representation of the agreement between the two- and multiple-point methods.

## Results

### Test-retest reliability of force and velocity

As shown in Table 1, the ICCs and CVs for the mean force were very high (excellent) under all load conditions. Regarding the mean velocity, the ICCs were very high (excellent) for the 100%–140%, 180%, 200%, 260%, and 280% BW load conditions, and high (good) for the 160%, 220%, 240%, and 300% BW load conditions. The CVs were excellent for the 100%–280% BW load conditions and good for the 300% BW load condition. The SEMs were 0.1–0.2 N/kg for the mean force and 0.0–0.1 m/s for the mean velocity.

**Table 1:** Mean force and mean velocity in 11 different loads (mean ± SD, n = 16) relative to body weight (100%–300% BW) and their reliability based on intraclass correlation coefficient (ICC), coefficient of variation (CV), and standard error of measurement (SEM).

	Mean force (N/kg)				Mean velocity (m/s)			
Load (% BW)	Mean ± SD	ICC (95% CI)	CV (95% CI)	SEM	Mean ± SD	ICC (95% CI)	CV (95% CI)	SEM
100	15.4 ± 1.0	0.98 (0.93 - 0.99)	0.9 (0.6 - 1.3)	0.2	1.2 ± 0.1	0.95 (0.88 - 0.98)	1.5 (1.1 - 2.3)	0.0
120	16.9 ± 1.0	0.99 (0.96 - 1.00)	0.6 (0.5 - 1.0)	0.1	1.2 ± 0.1	0.96 (0.89 - 0.99)	1.6 (1.2 - 2.5)	0.0
140	18.4 ± 0.9	0.95 (0.86 - 0.98)	0.9 (0.6 - 1.3)	0.2	1.1 ± 0.1	0.91 (0.75 - 0.97)	2.1 (1.6 - 3.3)	0.0
160	20.0 ± 0.9	0.95 (0.86 - 0.98)	0.8 (0.6 - 1.3)	0.2	1.0 ± 0.1	0.90 (0.74 - 0.96)	2.5 (1.8 - 3.8)	0.0
180	21.4 ± 0.9	0.97 (0.93 - 0.99)	0.5 (0.4 - 0.8)	0.1	0.9 ± 0.1	0.93 (0.82 - 0.98)	1.9 (1.4 - 3.0)	0.0
200	23.0 ± 0.9	0.98 (0.94 - 0.99)	0.5 (0.4 - 0.8)	0.1	0.9 ± 0.1	0.94 (0.84 - 0.98)	2.2 (1.6 - 3.4)	0.0
220	24.6 ± 0.9	0.95 (0.87 - 0.98)	0.5 (0.4 - 0.8)	0.2	0.8 ± 0.1	0.90 (0.73 - 0.96)	2.3 (1.7 - 3.5)	0.0
240	26.2 ± 0.8	0.96 (0.87 - 0.99)	0.6 (0.4 - 0.9)	0.2	0.8 ± 0.1	0.87 (0.65 - 0.95)	3.3 (2.4 - 5.1)	0.0
260	27.7 ± 0.8	0.96 (0.90 - 0.99)	0.5 (0.3 - 0.7)	0.2	0.7 ± 0.1	0.94 (0.84 - 0.98)	2.7 (2.0 - 4.2)	0.0
280	29.4 ± 0.8	0.98 (0.96 - 0.99)	0.3 (0.2 - 0.5)	0.1	0.6 ± 0.1	0.90 (0.75 - 0.97)	4.1 (3.0 - 6.3)	0.0
300	31.0 ± 0.8	0.94 (0.84 - 0.98)	0.5 (0.4 - 0.8)	0.2	0.5 ± 0.1	0.77 (0.46 - 0.91)	7.6 (5.6 - 11.7)	0.1

### Comparison between the two-point methods and the multiple-point method

Table 2 compares the multiple-point method and the ten two-point methods for the F–V parameters. Two-point methods based on closer loads (e.g., 100% and 120%, 100% and 140%, 100% and 160% BW) tended to overestimate F0 and Pmax, particularly when compared with methods using more separated loads (e.g., 100% and 180%, 100% and 300%), which produced values similar to those obtained with the multiple-point method. In fact, the bias when two-point methods based on distant loads (e.g., 100% and 180%, 100% and 300% BW) were compared to the multiple-point method was good (mean percent bias < 5%) for all F–V parameters.

**Table 2:** Force–velocity (F–V) parameters (mean ± SD, n = 16) such as theoretical maximum force (F0), velocity (V0), power (Pmax), and slope of the linear F–V relationship (SFv) obtained from the multiple-point method and 10 two-point methods using a combination of two different loads (100% and 120%, 100% and 140%, 100% and 160%, 100% and 180%, 100% and 200%, 100% and 220%, 100% and 240%, 100% and 260%, 100% and 280%, and 100% and 300% of body weight [BW]) in leg press exercise.

F–V method	F0 (N/kg)	V0 (m/s)	Pmax (W/kg)	SFv (N·s/m/kg)
Multiple-point	42.96 ± 3.26	1.90 ± 0.21	20.40 ± 2.77	−22.90 ± 3.30
Two-point 100% and 120%	53.26 ± 26.23	1.86 ± 0.34	23.65 ± 9.37	−30.73 ± 18.39
100% and 140%	48.05 ± 12.29	1.88 ± 0.35	21.75 ± 2.94	−27.56 ± 12.47
100% and 160%	47.00 ± 10.70	1.87 ± 0.30	21.43 ± 3.01	−26.52 ± 10.73
100% and 180%	43.34 ± 5.56	1.91 ± 0.28	20.53 ± 2.60	−23.38 ± 6.21
100% and 200%	43.54 ± 5.26	1.90 ± 0.25	20.58 ± 2.81	−23.47 ± 5.57
100% and 220%	43.54 ± 4.18	1.90 ± 0.24	20.56 ± 2.79	−23.43 ± 4.52
100% and 240%	43.80 ± 3.99	1.89 ± 0.24	20.60 ± 2.76	−23.65 ± 4.36
100% and 260%	43.34 ± 3.47	1.89 ± 0.23	20.50 ± 2.79	−23.24 ± 3.78
100% and 280%	43.58 ± 3.58	1.89 ± 0.22	20.56 ± 2.87	−23.43 ± 3.66
100% and 300%	43.05 ± 3.45	1.90 ± 0.22	20.44 ± 2.86	−22.97 ± 3.44

**Fig 2:**
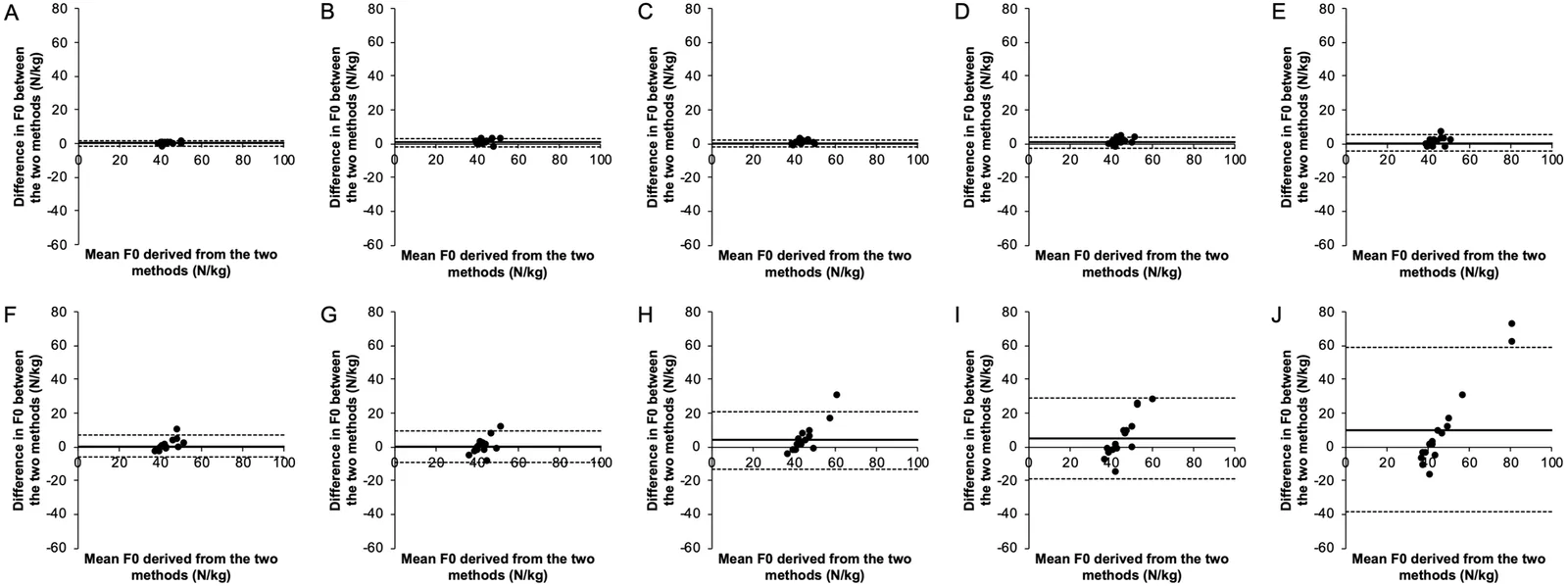
Bland–Altman plots showing the agreement of the theoretical maximum force (F0) obtained from the 10 two-point methods using two loads: (100% and 300% [A], 100% and 280% [B], 100% and 260% [C], 100% and 240% [D], 100% and 220% [E], 100% and 200% [F], 100% and 180% [G], 100% and 160% [H], 100% and 140% [I], and 100% and 120% [J] of body weight [BW]) with that obtained from the multiple-point method during leg press exercise.

**Fig 3:**
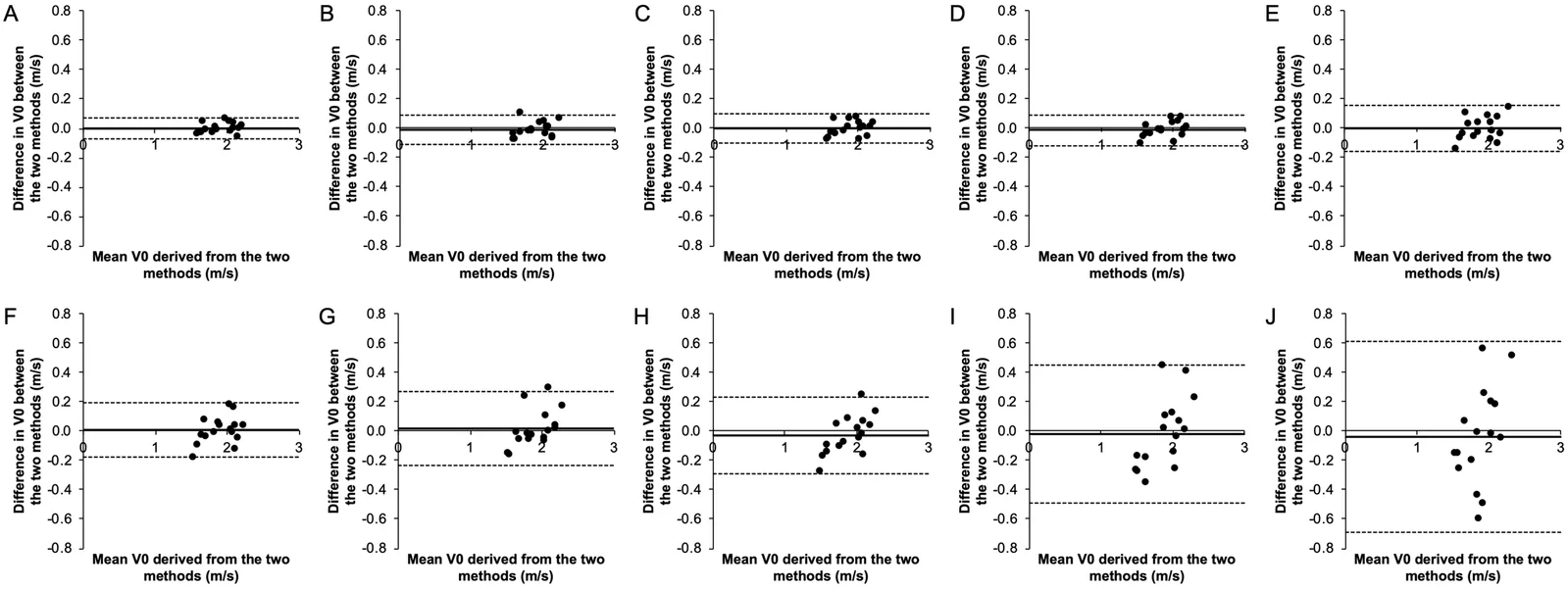
Bland–Altman plots showing the agreement of the theoretical maximum velocity (V0) obtained from the 10 two-point methods using two loads: (100% and 300% [A], 100% and 280% [B], 100% and 260% [C], 100% and 240% [D], 100% and 220% [E], 100% and 200% [F], 100% and 180% [G], 100% and 160% [H], 100% and 140% [I], and 100% and 120% [J] of body weight [BW]) with that obtained from the multiple-point method during leg press exercise.

**Fig 4:**
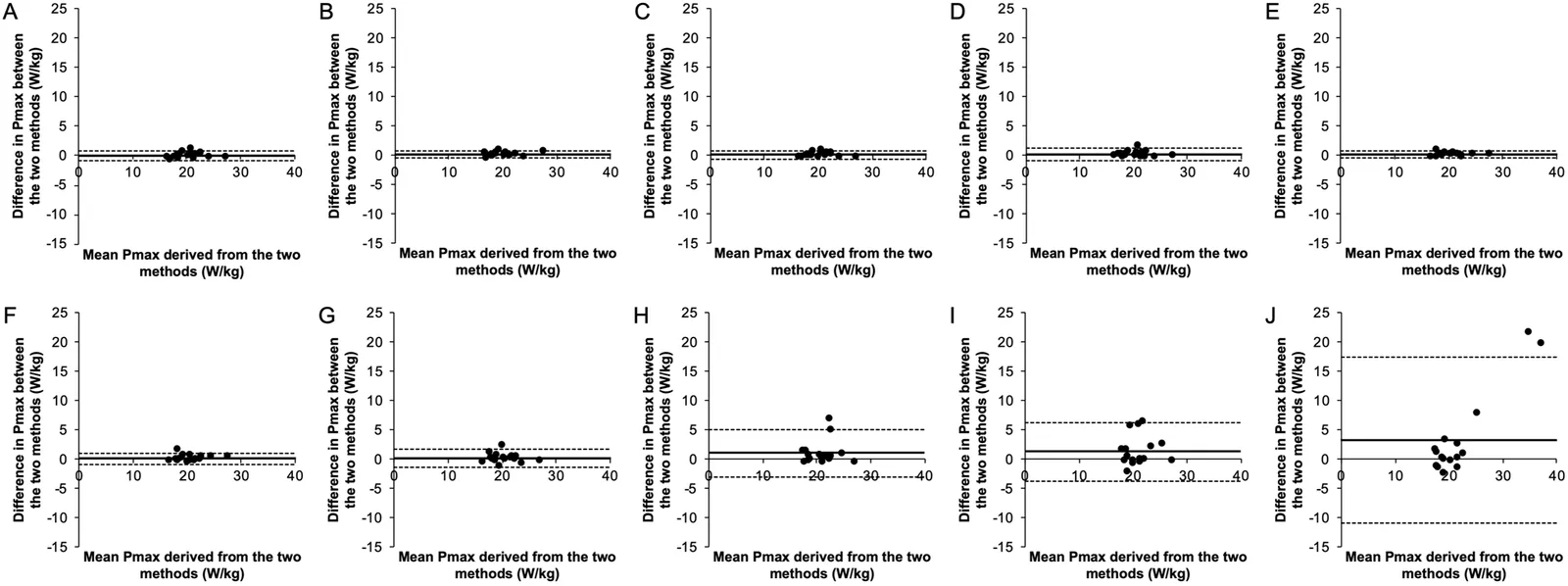
Bland–Altman plots showing the agreement of the theoretical maximum power (Pmax) obtained from the 10 two-point methods using two loads: (100% and 300% [A], 100% and 280% [B], 100% and 260% [C], 100% and 240% [D], 100% and 220% [E], 100% and 200% [F], 100% and 180% [G], 100% and 160% [H], 100% and 140% [I], and 100% and 120% [J] of body weight [BW]) with that obtained from the multiple-point method during leg press exercise.

**Fig 5:**
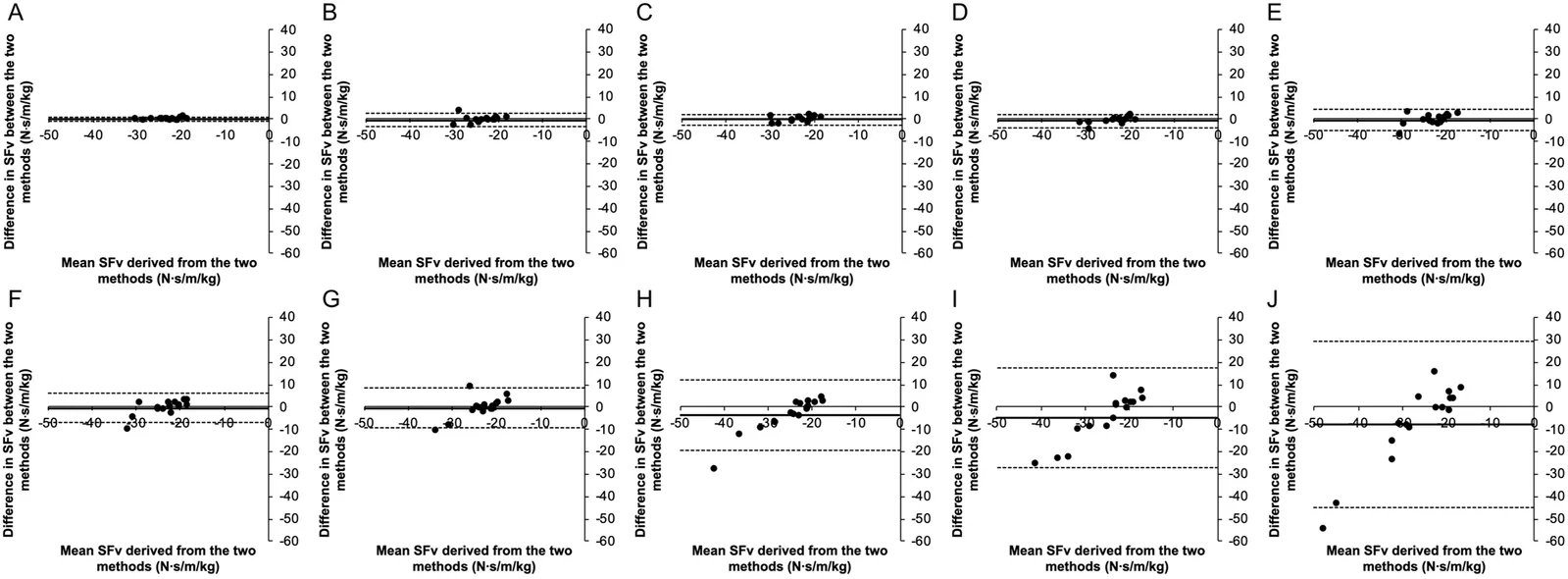
Bland–Altman plots showing the agreement of the slope of the linear F–V relationship (SFv) obtained from the 10 two-point methods using two loads: (100% and 300% [A], 100% and 280% [B], 100% and 260% [C], 100% and 240% [D], 100% and 220% [E], 100% and 200% [F], 100% and 180% [G], 100% and 160% [H], 100% and 140% [I], and 100% and 120% [J] of body weight [BW]) with that obtained from the multiple-point method during leg press exercise.

The F–V relationship based on the multiple-point method was highly linear when obtained from the individual force and velocity data (*r* = 0.974 to 0.998). Fig 1 shows the F–V relationships of the three participants with different F–V profiles. The F–V relationships obtained by the two-point method with two closer loads (i.e., 100% and 120%, 100% and 140%, and 100% and 160% BW) were different from those obtained by the multiple-point method. The four two-point methods with more separated loads (i.e., 100% and 240%, 100% and 260%, 100% and 280%, and 100% and 300% BW) provided nearly perfect correlations (*r* = 0.906 to 0.993) and trivial to small differences in magnitudes (Cohen’s *d* = −0.193 to 0.229) compared with the multiple-point method for all F–V parameters (Table 3). Bland–Altman plots (Figs 2–5) demonstrated that the two-point method using more distant loads showed better agreement with the multiple-point method, whereas the two-point method using closer loads showed poorer agreement.

**Table 3:** Difference in the force–velocity (F–V) relationship parameters such as theoretical maximum force (F0), velocity (V0), power (Pmax), and slope of the linear F–V relationship (SFv) obtained from the 10 two-point methods using two loads (100% and 120%, 100% and 140%, 100% and 160%, 100% and 180%, 100% and 200%, 100% and 220%, 100% and 240%, 100% and 260%, 100% and 280%, and 100% and 300% of body weight [BW]) in comparison to the multiple-point method during leg press exercise. Comparison to the multiple-point method is shown by *P*-value, Cohen’s *d,* Pearson’s *r* (95% confidence interval [CI]), and Bias (95% CI).

Two-point method	Parameter	*P*-value	Cohen’s *d*	*r* (95% CI)	Bias (95% CI)
100% and 120%	F0 (N/kg)	0.120	0.551	0.43 (−0.08 - 0.77)	−10.31 (−23.62 - 3.00)
	V0 (m/s)	0.650	−0.137	0.28 (−0.25 - 0.68)	0.03 (−0.15 - 0.21)
	Pmax (W/kg)	0.091	0.471	0.84 (0.59 - 0.94)	−3.26 (−7.09 - 0.58)
	SFv (N·s/m/kg)	0.117	−0.593	−0.06 (−0.54 - 0.45)	7.81 (−2.24 - 17.86)
100% and 140%	F0 (N/kg)	0.121	0.565	0.11 (−0.41 - 0.58)	−5.07 (−11.66 - 1.52)
	V0 (m/s)	0.759	−0.065	0.71 (0.33 - 0.89)	0.02 (−0.11 - 0.15)
	Pmax (W/kg)	0.051	0.473	0.60 (0.16 - 0.85)	−1.35 (−2.71 - 0.01)
	SFv (N·s/m/kg)	0.121	−0.510	0.46 (−0.04 - 0.78)	4.64 (−1.39 - 10.67)
100% and 160%	F0 (N/kg)	0.086	0.510	0.69 (0.30 - 0.89)	−4.04 (−8.70 - 0.63)
	V0 (m/s)	0.380	−0.116	0.90 (0.72 - 0.96)	0.03 (−0.04 - 0.10)
	Pmax (W/kg)	0.062	0.359	0.75 (0.40 - 0.91)	−1.04 (−2.14 - 0.06)
	SFv (N·s/m/kg)	0.090	−0.456	0.88 (0.67 - 0.96)	3.61 (−0.65 - 7.88)
100% and 180%	F0 (N/kg)	0.751	0.083	0.53 (0.05 - 0.81)	−0.38 (−2.89 - 2.13)
	V0 (m/s)	0.645	0.061	0.86 (0.64 - 0.95)	−0.01 (−0.09 - 0.07)
	Pmax (W/kg)	0.494	0.051	0.96 (0.88 - 0.99)	−0.13 (−0.55 - 0.30)
	SFv (N·s/m/kg)	0.681	−0.096	0.70 (0.31 - 0.89)	0.46 (−1.98 - 2.89)
100% and 200%	F0 (N/kg)	0.488	0.132	0.81 (0.53 - 0.93)	−0.58 (−2.30 - 1.15)
	V0 (m/s)	0.861	0.018	0.89 (0.71 - 0.96)	0.00 (−0.06 - 0.06)
	Pmax (W/kg)	0.160	0.065	0.99 (0.96 - 1.00)	−0.19 (−0.45 - 0.07)
	SFv (N·s/m/kg)	0.504	−0.124	0.84 (0.59 - 0.94)	0.56 (−1.21 - 2.33)
100% and 220%	F0 (N/kg)	0.342	0.153	0.83 (0.57 - 0.94)	−0.57 (−1.81 - 0.67)
	V0 (m/s)	0.866	−0.015	0.93 (0.81 - 0.98)	0.01 (−0.04 - 0.05)
	Pmax (W/kg)	0.052	0.058	0.99 (0.98 - 1.00)	−0.14 (−0.31 - 0.02)
	SFv (N·s/m/kg)	0.407	−0.133	0.84 (0.60 - 0.95)	0.51 (−0.81 - 1.83)
100% and 240%	F0 (N/kg)	0.053	0.229	0.92 (0.79 - 0.97)	−0.83 (−1.68 - 0.01)
	V0 (m/s)	0.366	−0.056	0.97 (0.90 - 0.99)	0.01 (−0.02 - 0.05)
	Pmax (W/kg)	0.126	0.073	0.98 (0.95 - 0.99)	−0.20 (−0.47 - 0.07)
	SFv (N·s/m/kg)	0.067	−0.193	0.96 (0.89 - 0.99)	0.74 (−0.07 - 1.54)
100% and 260%	F0 (N/kg)	0.170	0.113	0.95 (0.87 - 0.98)	−0.36 (−0.93 - 0.20)
	V0 (m/s)	0.714	−0.022	0.96 (0.88 - 0.99)	0.01 (−0.03 - 0.04)
	Pmax (W/kg)	0.226	0.038	0.99 (0.98 - 1.00)	−0.11 (−0.29 - 0.07)
	SFv (N·s/m/kg)	0.293	−0.094	0.95 (0.86 - 0.98)	0.31 (−0.34 - 0.97)
100% and 280%	F0 (N/kg)	0.101	0.182	0.92 (0.78 - 0.97)	−0.62 (−1.37 - 0.13)
	V0 (m/s)	0.416	−0.050	0.96 (0.88 - 0.99)	0.01 (−0.03 - 0.04)
	Pmax (W/kg)	0.082	0.057	0.99 (0.98 - 1.00)	−0.14 (−0.33 - 0.05)
	SFv (N·s/m/kg)	0.194	−0.150	0.91 (0.74 - 0.97)	0.52 (−0.31 - 1.34)
100% and 300%	F0 (N/kg)	0.621	0.026	0.98 (0.94 - 0.99)	−0.09 (−0.46 - 0.29)
	V0 (m/s)	0.939	0.003	0.97 (0.91 - 0.99)	0.01 (−0.02 - 0.04)
	Pmax (W/kg)	0.692	0.016	0.99 (0.96 - 1.00)	−0.04 (−0.29 - 0.21)
	SFv (N·s/m/kg)	0.644	−0.019	0.99 (0.96 - 1.00)	0.06 (−0.24 - 0.35)

## Discussion

This study tested the hypothesis that the concurrent validity of the F–V relationship parameters obtained from the two-point method relative to the multiple-point method is high when different loads are used, whereas this validity progressively decreases when closer loads are used. The results indicate that only four two-point methods using different loads (100% and 240%, 100% and 260%, 100% and 280%, and 100% and 300% BW) provided nearly perfect correlations with the F–V relationship obtained from the multiple-point method and showed trivial to small differences and acceptable bias (<5%) for field use in all F–V parameters compared with those from the multiple-point method. In contrast, the concurrent validity of the F–V relationship parameters obtained from the two-point methods relative to the multiple-point method tended to decrease with the use of loads close to each other (e.g., 100% and 120% BW). These results support our hypotheses and indicate that the two-point method using distant loads yields leg press F–V relationship parameters that are well matched to those from the multiple-point method.

Reliable F–V profiling under multiple load conditions requires that the force and velocity of each input data point be highly reliable (ICC > 0.75, CV < 10%) [20,26,28]. The ICCs and CVs for mean force were excellent under all load conditions (Table 1). Regarding mean velocity, excellent reliability was demonstrated for the ICC and/or CV under the 280% BW load condition and below. However, reliability decreased under heavier load conditions (i.e., 300% BW). Previous studies [29–31] demonstrated that increased external load reduces the reliability of velocity-related indicators. García-Ramos et al. [31] reported that the reliability of cadence and power values tended to decrease as the resistive forces increased during cycle ergometer testing. Pérez-Castilla et al. [29,30] showed that the CVs of the squat jump and countermovement jump height increased to an unacceptable level (>10%) under load conditions when the jump height fell below 10 cm. In this study, the mean velocity was 0.52 m/s under the 300% BW load condition, where a decline in reliability was observed (Table 1). A plausible mechanism for the velocity-specific reduction in reliability at the heaviest external load condition is the increased sensitivity of velocity calculation to between-trial movement variability when participants approach their maximal force capacity. A recent systematic review of 22 biomechanical studies in experienced lifters reported that joint coordination and segmental variability consistently increased with intensity, and that individual differences in movement strategy became more pronounced at higher loads [32]. Although these findings were derived from free-weight exercises with greater kinematic degrees of freedom than the present horizontal leg press device, between-trial variability, such as the timing of force initiation, left–right leg synchronization, and the consistency of completion time used in the velocity calculation, is likely to persist even with a fixed seat and standardized starting knee angle. At 300% BW, where mean velocity fell to approximately 0.5 m/s, and participants were operating close to their force ceiling, even modest absolute variability in completion time would translate into proportionally large variability in calculated mean velocity. By contrast, force at the same condition was integrated across the entire range of motion and was less affected by such timing-related variability, which is consistent with the preserved reliability of force across all external load conditions in the present study. These findings suggest that data points obtained under very high load conditions, where the mean velocity falls below approximately 0.5 m/s, should be excluded to enhance the reliability of leg press F–V profiling.

The F–V relationship parameters obtained using the two-point method with more distinct loads showed better agreement with those obtained using the multiple-point method (Tables 2 and 3). These results were consistent with the findings of a previous study [20] on vertical jumps, which showed that the two-point method using different loads exhibited high concurrent validity in comparison to the multiple-point method. This study provides further support for the validity of the two-point method using distant loads in lower-limb multi-joint tasks. Furthermore, the F–V relationship in the leg press task in the present study was highly linear (*r* = 0.974–0.998), as shown in Fig 1. The high linearity of the F–V relationship allowed several two-point methods with significantly different data points (i.e., 100% and 240%, 100% and 260%, 100% and 280%, and 100% and 300% BW) to demonstrate comparably high concurrent validity with the multiple-point method. García-Ramos et al. [20] also reported for vertical jumps that two two-point methods based on relatively distant loads (i.e., 0 and 60 kg and 0 and 75 kg) exhibited equivalent concurrent validity with the multiple-point method. Considering these findings, it is important to use two-point methods based on data points separated by a sufficient distance for accurate measurement; however, it may not be necessary to use the two most distant points.

The concurrent validity of the F–V relationship parameters obtained using the two-point method relative to the multiple-point method tended to decrease with the use of more closer loads (Fig 1, Table 3). As this trend was more pronounced in F0 than in V0, the reduced concurrent validity of Pmax and SFv with the multiple-point method was thought to be caused primarily by F0 rather than V0. This effect may result from estimating the F–V relationship parameters using data extrapolation. In this study, the applied lightest load (i.e., 100% BW) was fixed for all two-point methods, consistent with prior research [20], whereas the applied heaviest load varied among the methods, ranging from 120% to 300% BW. Consequently, the two-point method using two closely spaced loads (i.e., 100% and 120% BW) relies on relatively velocity-biased data points (Fig 1, Table 1). This method likely increased the extrapolation errors [6,18,33] of F0 and decreased the concurrent validity with the multiple-point method. Thus, minimizing the extrapolation errors in the two-point method requires using two more widely separated loads.

This study, together with previous studies [18,20], demonstrated the usefulness of the two-point method for estimating the F–V profile. However, some limitations exist. This study involved relatively strong young men who were capable of completing tests under external load conditions of 100–300% BW imposed by a horizontal dynamic leg press device. Therefore, it remains unclear whether the findings of the present study apply to other populations, including women and older adults, who may be unable to complete leg press tasks under high-load conditions. A further limitation pertains to the loading design of the two-point methods. In the present study, all two-point methods were anchored at 100% BW as the lowest load, while only the heaviest load was varied from 120% to 300% BW. As a consequence of this design, the present findings identify the best-performing two-point loading combinations among those anchored at 100% BW, rather than the globally optimal two-point loading strategy across all possible anchor-load combinations. It remains unknown whether two-point methods using a lower anchor load (e.g., 60% or 80% BW), a higher anchor load, or load pairs not anchored at body weight would yield comparable, superior, or inferior agreement with the multiple-point reference method. Future studies should systematically examine how varying both the lower and upper anchor loads affects the agreement of two-point F–V profiling in horizontal dynamic leg press exercises. Additionally, the current study used a horizontal dynamic leg press device equipped with pneumatic artificial muscles, which differs somewhat from other leg press machines. This device was selected in the present study because the validity of two-point F–V profiling could only be meaningfully evaluated on a measurement system providing force and velocity outputs with high test–retest reliability, since measurement variability propagates more strongly into estimates derived from only two experimental points than into those derived from a larger number of points [18]. This difference in loading type may limit the generalizability of the present study’s findings when other leg press devices are used because the measured F–V relationship changes depending on the loading type (e.g., pneumatic vs. isoinertial) [34]. However, based on previous studies [6,15,35,36] indicating that the F–V relationship for leg press tasks is quasi-linear across various load types, the principle that two-point methods using more distant loads provide more accurate F–V parameter estimates than methods using closer loads is expected to apply when leg press tasks are performed on other devices. This is grounded in the mathematics of linear regression rather than in device-specific properties [18]. However, the specific load combinations identified here may differ across devices and should be empirically confirmed on other systems. Finally, we investigated the concurrent validity of the two-point method using data obtained from a single session of the multiple-point method. However, using the two-point method under actual field conditions may produce higher values for some F–V relationship parameters because of reduced fatigue [37,38]. Future research should directly investigate whether fixed bias occurs when the two-point method is used for F–V profiling of the leg press task in an independent measurement session.

## Conclusions

The current study demonstrated that certain two-point methods are valid for testing maximal force, velocity, and power production capabilities during leg press movements. Specifically, using relatively distant loads (100% and 240%, 100% and 260%, 100% and 280%, and 100% and 300% BW) in the two-point method enables the acquisition of more valid F–V parameters and provides a time-efficient, fatigue-reducing alternative (e.g., 4 vs. 22 trials in the present study). Therefore, the two-point method using widely separated loads can serve as a practical alternative to the multiple-point method for assessing leg extension capability, at least in young, relatively strong men tested on a horizontal dynamic leg press equipped with pneumatic artificial muscles. Generalization to other populations and leg press devices warrants further investigation.

## Supporting information

S1 Dataset Original data. (XLSX)
