# Supplementary material for: Optimal two-point loading strategy for accurate leg press force–velocity assessment
Source: PLoS One. 2026 Aug 25;21(8):e0356566. doi: 10.1371/journal.pone.0356566 (PMC13505900; doi:10.1371/journal.pone.0356566)
Supplement: Supplementary file 1 [file pone.0356566.s003.docx]

Responses to Reviewers

Thank you for the opportunity to resubmit our manuscript entitled, “Optimal two-point loading strategy for accurate leg press force–velocity assessment.” We have carefully reviewed the reviewers’ comments and revised the manuscript accordingly. All necessary changes have been made, and detailed responses to each comment are provided below. We believe our responses are adequate, the revised manuscript (PONE-D-25-63455-R1) has been improved, and meets the criteria for publication in the journal.

**Reviewer #1**

1. The current analysis relies on Pearson’s r, paired t-tests, and Cohen’s d. While useful, Pearson’s r evaluates linear correlation rather than absolute agreement between two methods.

Response: We fully agree with your comment. In response to your feedback, we have added Bland–Altman plots (Figures 2–5) showing that the two-point method using more distant loads provides better agreement with the multiple-point method. The following sentences have been added to the manuscript:

“Bland–Altman plots were generated, providing a comprehensive representation of the agreement between the two- and multiple-point methods.” (lines 191–192)

“Bland–Altman plots (Figures 2–5) demonstrated that the two-point method using more distant loads showed better agreement with the multiple-point method, whereas the two-point method using closer loads showed poorer agreement.” (lines 232–235)

2. The authors should clarify whether a sample size of n=16 provides sufficient statistical power to detect meaningful differences in the validity assessments between the various two-point models.

Response: The sample size of 16 was determined a priori using G*Power (version 3.1.9.6) with the following parameters: an expected correlation coefficient of r = 0.7 (based on García-Ramos et al. [20]), a significance level of α = 0.05, and a statistical power (1 − β) of 0.95. As clarified in the revised “Participants” section (lines 93–97), this calculation was specifically designed to detect a significant correlation between the F–V relationship parameters obtained from the two-point and multiple-point methods, which was the main aim of this study. The chosen power level (0.95) is more stringent than the conventional 0.80, providing a substantial safeguard against Type II error in the validity assessments. Furthermore, the resulting sample size is comparable to those used in previous validation studies of the two-point method for lower-limb tasks (e.g., García-Ramos et al. [20]), further supporting its adequacy for the present validity analyses. In addition, we have noted in the Conclusion section that our sample represents a specific population (young, relatively strong men tested on a single device), which may limit the generalizability of our findings (lines 388–393).

3. The authors acknowledge the use of a pneumatic system as a limitation regarding generalizability. It would significantly strengthen the discussion to briefly explain why this matters from a biomechanical perspective.

Response: Thank you very much for the valuable suggestion. Previous research (Avrillon et al., 2017) on the ballistic bench press has reported that the measured force–velocity (F–V) relationship varies depending on the loading modality. Specifically, pneumatic resistance mitigates inertial influences compared to isoinertial resistance, thereby limiting the magnitude of the initial force required to displace the barbell. These findings suggest that the loading modality affects the F–V relationship, highlighting it as a critical factor to be considered in its measurement. Accordingly, while we have acknowledged the use of a pneumatic leg press device with artificial muscles as a limitation of the present study, it is important to note that the F–V relationship in leg press tasks has been consistently shown to follow a linear trend across various loading patterns. Based on this rationale, we have addressed the generalizability of our findings to other leg press devices in the revised Discussion. Furthermore, we have revised the manuscript to more clearly state our intentions behind addressing the loading modality as follows:

“This difference in loading type may limit the generalizability of the present study’s findings when other leg press devices are used because the measured F–V relationship changes depending on the loading type (e.g., pneumatic vs. isoinertial) [34].” (lines 369–371)

4. There is a duplication error in Table 2.

Response: Thank you very much for pointing that out. We have corrected the duplication error in Table 2.

**Reviewer #2**

General comment

I congratulate the authors on their efforts in conducting this study, which aimed to examine the concurrent validity of the F–V relationship during a horizontal leg press exercise by comparing two-point methods with a multiple-point method. The results indicated that the F–V relationship obtained from the four two-point methods with wider load differences (100% and 240%, 100% and 260%, 100% and 280%, and 100% and 300% BW) was nearly perfectly correlated with that obtained by the multiple-point method (r = 0.906-0.993). The concurrent validity of the F–V relationship parameters obtained from the two-point methods tended to decrease when using loads that were closer together (e.g., 100% and 120% BW) than loads with a larger difference (e.g., 100% and 240% BW). Although the article presents important content for journal readers, indicating that, on a horizontal leg press, a two-point method based on sufficiently distinct loads can provide an accurate estimate of the F–V relationship and may be a practical alternative to comprehensive multiple-load testing, it has some clear limitations: a small sample size and the use of a pneumatic leg press machine limit the generalizability of the findings. Furthermore, the study’s practical applications should be reinforced. Therefore, the manuscript needs further clarification.

Below, I provide some suggestions.

Response: We sincerely thank the reviewer for their careful evaluation and for noting that our study provides important content for journal readers. We particularly appreciate the accurate summary of our key finding, namely that the two-point methods using more widely separated loads (100% and 240–300% BW) yielded F–V parameters that were nearly perfectly correlated with those obtained from the multiple-point method (r = 0.906–0.993).

We also acknowledge the three concerns raised in the overall assessment: (i) the sample size, (ii) the use of a pneumatic leg press machine, and (iii) the need to reinforce the practical applications. We address these concerns as follows and address each specific suggestion in the point-by-point replies below.

(i) The sample size of 16 was determined a priori using G*Power (expected r = 0.7 based on García-Ramos et al. [20], α = 0.05, power = 0.95), as described in the Participants section. This sample size is comparable to those reported in previous validation studies of the two-point method for lower-limb tasks (e.g., García-Ramos et al. [20]). In addition, we have noted in the Conclusion section that our sample represents a specific population, which may limit the generalizability of our findings (lines 391–395).

(ii) We agree that the use of a pneumatic horizontal dynamic leg press device (ddrobotec® System ELITE) is a device-specific feature of our study. As noted in the Discussion (lines 372–378), however, the quasi-linear nature of the F–V relationship in leg press tasks has been consistently reported across different loading modalities, which provides a theoretical basis for expecting that the two-point method can also be applied to other leg press devices.

(iii) We fully agree that the practical applications need to be addressed better. In the revised manuscript, we have expanded this section to include: (a) a concrete recommendation of optimal load pairs (100% combined with 240–300% BW) supported by our concurrent validity results; and (b) a quantification of the time- and fatigue-saving advantage (4 trials versus 22 trials in the present study) that the two-point method offers in field and clinical settings.

“Specifically, using relatively distant loads (100% and 240%, 100% and 260%, 100% and 280%, and 100% and 300% BW) in the two-point method enables the acquisition of more valid F–V parameters and provides a time-efficient, fatigue-reducing alternative (e.g., 4 vs. 22 trials in the present study).” (lines 388–395)

Introduction

Page 4, LL44: “Leg extensions are performed during various athletic activities...”

I suggest that authors provide examples of these activities.

Response: As you suggested, we have revised the sentence as follows:

“Leg extensions are performed during various athletic activities, such as vertical jumps or sprint running [1,2], and horizontal dynamic leg press exercises are widely used to train and evaluate leg extension performance [1,3–8].” (lines 45–47)

Page 4, LL46: “We [1] recently reported high reliability…”

I recommend that authors write the beginning of the sentence more formally. For example, you might consider beginning with: “Our research team recently reported.”

Response: As you suggested, we have revised the sentence as follows:

“Our research team recently reported high reliability in measuring force, velocity, and power during the horizontal dynamic leg press exercise using a device equipped with pneumatic artificial muscles (ddrobotec® System ELITE mk4, Dynamic Devices, Zurich, Switzerland), with some variables showing higher reliability than those measured in vertical jumps [1].” (lines 47–51)

Materials and Methods

Participants

Page 6, LL90-91: “The sample size was calculated using the G*Power software…”

The study included a sample size calculation, thereby providing added value.

Response: Thank you for your comments.

Page 6, LL92-94: “Based on an expected correlation coefficient of 0.7, a significance level of 0.05, and a statistical power of the required sample size was calculated to be 16 participants.”

The authors should support the sample size calculation with appropriate references.

Response: As you suggested, we have added the appropriate reference supporting the sample size calculation as follows:

“Based on an expected correlation coefficient of 0.7 [20], a significance level of 0.05, and a statistical power of 0.95, the required sample size was calculated to be 16 participants.” (lines 100–101)

Testing procedures

Page 7, LL113-115: “The rest periods progressively increased with increasing load; for the first three load levels, the rest intervals ranged from 10 to 20 seconds, and for the remaining loads, from 20 to 40 seconds.”

What evidence do the authors use to support these rest periods?

Response: In response to your comment, we have added previous studies that support the rest periods used in our study when using a horizontal leg press machine.

“The rest periods progressively increased with increasing load; for the first three load levels, the rest intervals ranged from 10 to 20 seconds, and for the remaining loads, from 20 to 40 seconds [1,5,6].” (lines 123–125)

Page 7, LL116-118: “Pneumatic semi-isotonic resistance prevented maximal effort from producing ballistic action, and the entire push-off was performed with maximal intentional velocity.”

Please, add references.

Response: As you suggested, we have added some references.

“Pneumatic semi-isotonic resistance prevented maximal effort from producing ballistic action, and the entire push-off was performed with maximal intentional velocity [1,5,6].” (lines 126–128)

Data analyses

Page 8, LL137: Have multiple regression analyses been considered for this analysis?

Response: This regression analysis was conducted to model the force–velocity relationship during the leg press exercise. It is known that the force–velocity relationship in multi-joint movements such as the leg press follows a quasi-linear pattern. Therefore, rather than using multiple regression analysis, we conducted a least-squares linear regression, as recommended in previous studies [6,20]. Thus, we believe that this analysis was appropriate.

Statistical analyses

Page 10, LL137: Measurement reliability.

I recommend that the authors separate the reliability analysis into two parts for improved accuracy: relative reliability (ICC) and absolute reliability (CV and SEM).

Response: In response to your feedback, we have revised the sentence as follows:

“The intraclass correlation coefficient (ICC; two-way mixed effects, absolute agreement, single measurement) [24] was calculated to analyze relative reliability, and the coefficient of variation (CV) and standard error of measurement (SEM) [25] were calculated to analyze absolute reliability.” (lines 175–178)

Results

The results subsection is well-articulated and accurately presented.

Response: Thank you very much.

Discussion

Page 15, LL225-227: “This study tested the hypothesis that the concurrent validity of the F–V relationship parameters obtained from the two-point method is high when different loads are used, whereas this validity progressively decreases when closer loads are used.”

I recommend that the authors revise the paragraph to enhance its clarity and overall comprehension of the study’s theme.

Response: As you recommended, we have revised the sentence to improve the clarity and highlight findings related to the main topic of the study in the last sentence of the paragraph.

“These results support our hypotheses and indicate that the two-point method using distant loads yields leg press F–V relationship parameters that are well matched to those from the multiple-point method.” (lines 285–287)

Page 16, LL233-234: “These results support our hypotheses.”

The sentence appears isolated; the authors should integrate the idea into another sentence within the paragraph.

Response: As you pointed out, that sentence is too short and isolated. However, combining it with other sentences risks making the sentence too long or causing it to deviate from the original point. Therefore, we have expanded the sentence as mentioned above (lines 285–287).

Page 16, LL235-236: “Reliable F–V profiling under multiple load conditions requires that the force and velocity of each input data point be highly reliable.”

I recommend that authors include reference values to clarify which metrics can be deemed highly reliable.

Response: As you suggested, we have included reference values in the sentence.

“Reliable F–V profiling under multiple load conditions requires that the force and velocity of each input data point be highly reliable (ICC > 0.75, CV < 10%) [20,26].” (lines 288–289)

Page 16, LL246-248: “These findings suggest that data points obtained under very high load conditions, where the mean velocity falls below approximately 0.5 m/s, should be excluded to enhance the reliability of leg press F–V profiling.”

The authors should explore in greater depth the factors that contribute to the diminished reliability of velocity-related indicators under very high load conditions.

Response: We agree that the original Discussion noted the empirical pattern but did not adequately explore its underlying mechanism. We have added the following explanations to the Discussion proposing that the velocity-specific reduction in reliability observed at 300% BW reflects increased sensitivity of the velocity calculations to between-trial variability in movement timing—particularly the timing of force initiation, left–right leg synchronization, and consistency of completion time—when participants are operating close to their maximal force capacity. We support this argument with reference to a recent systematic review (Bakhshinejad et al., 2025, Sports Med Open) that synthesized 22 biomechanical studies and concluded that joint coordination and segmental variability consistently increase with exercise intensity in experienced lifters. We also explicitly acknowledge that those previous findings were obtained with free-weight exercises having greater kinematic degrees of freedom than the present horizontal leg press device and clarify that between-trial variability persists even with the device’s mechanical standardization. The added paragraph also explicitly contrasts this velocity-specific phenomenon with the consistently excellent reliability of force across all external load conditions in our data.

“A plausible mechanism for the velocity-specific reduction in reliability at the heaviest external load condition is the increased sensitivity of velocity calculation to between-trial movement variability when participants approach their maximal force capacity. A recent systematic review of 22 biomechanical studies in experienced lifters reported that joint coordination and segmental variability consistently increased with intensity, and that individual differences in movement strategy became more pronounced at higher loads [32]. Although these findings were derived from free-weight exercises with greater kinematic degrees of freedom than the present horizontal leg press device, between-trial variability, such as the timing of force initiation, left–right leg synchronization, and the consistency of completion time used in the velocity calculation, is likely to persist even with a fixed seat and standardized starting knee angle. At 300% BW, where mean velocity fell below 0.5 m/s, and participants were operating close to their force ceiling, even modest absolute variability in completion time would translate into proportionally large variability in calculated mean velocity. By contrast, force at the same condition was integrated across the entire range of motion and was less affected by such timing-related variability, which is consistent with the preserved reliability of force across all external load conditions in the present study.” (lines 300–315)

Conclusion

Page 19, LL299-300: “Specifically, applying relatively distant loads in the two-point method enables the acquisition of more valid F–V parameters.”

I suggest that the authors include examples of the load values.

Response: As you suggested, we have included examples of the load values in the sentence.

“Specifically, using relatively distant loads (100% and 240%, 100% and 260%, 100% and 280%, and 100% and 300% BW) in the two-point method enables the acquisition of more valid F–V parameters and provides a time-efficient, fatigue-reducing alternative (e.g., 4 vs. 22 trials in the present study).” (lines 388–391)

**Reviewer #3**

Overall assessment

This manuscript addresses a relevant applied question in sports science: whether a simpler two-point loading method can provide a valid estimate of the force–velocity (F–V) profile during horizontal leg press exercise, compared with a more time-consuming multiple-point approach. The study is focused, the design is clear, and the main practical message is potentially useful. The results suggest that two-point methods using more widely separated loads perform better than methods using closer loads, and that some two-point combinations may approximate the multi-point method reasonably well.

The manuscript has merit and could become publishable after revision. However, several points need stronger treatment before acceptance. The main issues concern the framing of validity, the agreement analysis, the limited generalizability of the findings, and especially the device-specific nature of the resistance used. The practical implications of using a pneumatic semi-isotonic leg press rather than a conventional plate-loaded machine are not explained well enough.

Response: We sincerely thank the Reviewer for the constructive assessment and for the comment on the merit of the manuscript. We agree that each of the four main issues identified, namely the framing of validity, the agreement analysis, the limited generalizability of the findings, and the device-specific nature of the resistance used, should be addressed. We have revised the relevant sections of the manuscript. The specific revisions and their rationale are described in detail in our point-by-point responses shown below. We are grateful for the Reviewer’s careful reading and constructive guidance, which have materially strengthened the precision and scope of the manuscript’s claims.

Summary of the manuscript

The study compares ten two-point approaches for estimating leg press F–V parameters with a multiple-point method based on eleven loads from 100% to 300% body weight. Sixteen young men performed maximal horizontal leg press actions on a device equipped with pneumatic artificial muscles. The authors calculated F0, V0, Pmax, and slope, and compared the two-point estimates with the multiple-point estimates using paired tests, effect sizes, correlations, and bias. The main finding is that two-point methods using larger load separation, especially 100% paired with 240–300% body weight, showed the best agreement with the multiple-point method.

Strengths

The topic is practically important for both researchers and practitioners interested in reducing testing burden.

The study asks a clear question and tests multiple load pairings rather than only one candidate two-point approach.

The manuscript reports reliability metrics across loads, which helps readers judge measurement quality.

The main result is easy to interpret and likely useful in practice within the tested context.

The Discussion acknowledges some limitations, including population specificity and device dependence, although these parts need expansion.

Response: We are grateful to the Reviewer for the careful and constructive summary of our manuscript and for highlighting the strengths of the present study. We very much appreciate the Reviewer’s recognition of the practical relevance of the research question, the comprehensive testing of multiple load pairings, the inclusion of reliability metrics across loads, and the interpretability of the main findings within the tested context. With respect to the Reviewer’s note that the limitations regarding population specificity and device dependence would benefit from expansion, we fully agree, and we have expanded these limitations in the revised manuscript as shown below.

“Therefore, the two-point method using widely separated loads can serve as a practical alternative to the multiple-point method for assessing leg extension capability, at least in young, relatively strong men tested on a horizontal dynamic leg press equipped with pneumatic artificial muscles. Generalization to other populations and leg press devices warrants further investigation.” (lines 391–395)

Major comments

1. The reference method is not a true gold standard, so the validity claim should be framed more carefully,

The manuscript presents the analysis as a test of concurrent validity, but the multiple-point method is not an independent criterion method. It is another model-based estimate derived from the same session and the same measurement system. This makes the study a comparison against a broader-load reference approach rather than a validation against a true external standard.

Why this matters: the current wording risks overstating what has been shown.

What the authors should do: revise the wording throughout the manuscript to make clear that the study evaluates agreement or concurrent validity relative to a multi-point reference method, not criterion validity against an external gold standard. This is especially important in the Abstract, Discussion, and Conclusions.

Response: We fully accept the Reviewer’s point that the multiple-point method is not an independent external criterion, because both methods were derived from the same testing session and the same measurement system in the present study. We have followed the suggestion to clarify that the present study evaluated agreement or concurrent validity relative to a multi-point reference method in the Abstract, Discussion, and Conclusions.

(ⅰ) Explicit framing of “concurrent validity” throughout the manuscript: We have revised the manuscript throughout to ensure that every instance of “concurrent validity” is explicitly anchored to the multiple-point method as the reference, rather than left to imply a comparison against an external gold standard. At each occurrence where the comparator was previously implicit or absent, we have inserted explicit language such as “with the multiple-point reference method,” “relative to the multiple-point reference method,” or “in comparison to the multiple-point method,” so that no statement of validity in the manuscript stands without a clearly identified within-session reference. The specific locations of these revisions are at lines 80–81, 276, 283–284, 335, 337, and 344–345.

Together with the existing explicit framing in the Abstract, the Introduction purpose statement, and the Methods Experimental design subsection, the revised manuscript now consistently positions “concurrent validity” as the evaluation of agreement with the within-session multiple-point reference method, rather than as criterion validity against an external standard.

(ⅱ) Rationale for retaining the term “concurrent validity”: We respectfully note that the use of “concurrent validity” with the multiple-point method serving as the reference is a long-established terminological and methodological convention in the F–V profiling literature. The foundational review of the two-point method by García-Ramos [18], the seminal validation study in vertical jumps by García-Ramos et al. [20], and subsequent validation studies across bench press, bench pull, leg cycle ergometer, and other tasks have all adopted this framing. The recent comprehensive review by García-Ramos [18] explicitly summarizes this convention and, importantly, acknowledges precisely the limitation raised by the Reviewer (that obtaining data from the same test session may inflate the apparent concurrent validity) while continuing to adopt the same terminology, because the multiple-point method represents the de facto reference standard against which the two-point method has been progressively validated in this field. Retaining the term “concurrent validity” therefore preserves direct terminological comparability between our findings and the prior validation literature, while the revisions described in (ⅰ) above ensure that the comparator is never ambiguous to the reader.

(ⅲ) Disclosure of the within-session design in the existing Limitations section: We respectfully draw the Reviewer's attention to the closing sentences of the Limitations paragraph in the original manuscript, which describe precisely the design feature underlying the Reviewer's concern. Specifically, the existing text reads:

“Finally, we investigated the concurrent validity of the two-point method using data obtained from a single session of the multi-point method. However, using the two-point method under actual field conditions may produce higher values for some F–V relationship parameters because of reduced fatigue [37,38]. Future research should directly investigate whether fixed bias occurs when the two-point method is used for F–V profiling of the leg press task in an independent measurement session.” (lines 378–383)

This passage transparently states that (a) the two-point and multiple-point estimates in the present study were both derived from a single testing session of the multi-point measurement, (b) the agreement patterns reported here may therefore not be reproduced when the two-point method is implemented in a session independent of the multi-point reference, and (c) future work is required to verify whether systematic (fixed) bias emerges under such independent-session conditions. Taken together, these three statements openly communicate that the multiple-point method served as a within-session reference method, rather than as a session-independent external criterion, which is precisely the design feature the Reviewer wishes to be acknowledged. Together with the explicit anchoring of “concurrent validity” to the multiple-point reference method throughout the revised manuscript (point ⅰ above), and with the retention of the established F–V profiling terminology for direct comparability with the prior two-point method literature (point ⅱ above), we believe the existing Limitations text already conveys the design constraint identified by the Reviewer transparently to the readership.

2. Correlation is not sufficient to support interchangeability between methods

The statistical approach includes paired t-tests, effect sizes, Pearson correlations, and bias. This is useful, but correlation alone does not establish that two methods can be used interchangeably. A strong correlation can still coexist with practically important disagreement.

Why this matters: the paper’s practical claim is that a two-point method may replace a multi-point test. That requires a stronger agreement framework.

What the authors should do: add agreement-focused analysis for the preferred two-point methods, ideally Bland–Altman plots or a similar method-comparison approach. If the authors do not wish to add such analyses, the practical conclusions should be made more cautious.

Response: We fully agree that Pearson correlation analyses are not sufficient to support the interchangeability of two measurement methods. We have therefore followed the Reviewer’s recommendation and added an agreement-focused analysis based on Bland–Altman methodology, and we believe this has substantially strengthened the methodological foundation of the manuscript. Specifically, the following additions and revisions have been made.

“Bland–Altman plots were generated, providing a comprehensive representation of the agreement between the two- and multiple-point methods.” (lines 191–192)

“Bland–Altman plots (Figures 2–5) demonstrated that the two-point method using more distant loads showed better agreement with the multiple-point method, whereas the two-point method using closer loads showed poorer agreement.” (lines 232–235)

3. The sample size justification does not fully match the main study aim

The sample size calculation appears to be based on an expected correlation coefficient and is described as being performed to assess reliability. However, the main aim of this paper is comparison between two-point and multiple-point methods.

Why this matters: the sample size rationale should align with the primary study objective.

What the authors should do: clarify whether the current sample size calculation was intended for reliability, method comparison, or both. If the study was powered only for a reliability-type analysis, this should be stated as a limitation.

Response: The Reviewer has correctly identified an apparent inconsistency between the stated purpose of the sample size calculation (“to assess reliability”) and the input parameter actually used (an expected correlation coefficient of 0.7). We apologize for this misleading wording: the term “reliability” in the original text was an inadvertent error and should have read “correlation between the two-point and multiple-point methods.” The original sample size calculation was based on the primary statistical analysis of the present study (Pearson’s correlation between the F–V relationship parameters obtained from the two-point and multiple-point methods). Therefore, the sample size calculation was aligned with the primary objective of the method comparison. Accordingly, we have corrected the wording in the Participants section.

“This study was conducted in Japan from December 16, 2022, to March 31, 2024. The sample size was calculated using the G*Power software (version 3.1.9.6, Düsseldorf, Germany) to determine the number of participants required to detect a significant correlation between the F–V relationship parameters obtained from the two-point and multiple-point methods, which was the main aim of this study.” (lines 95–99)

4. The fixed use of 100% body weight as the lower load narrows the meaning of “optimal”

All two-point methods were anchored at 100% body weight, with only the heavier load varying. Therefore, the study does not identify the globally optimal two-point loading strategy. It identifies the best-performing strategy among methods anchored at 100% body weight.

Why this matters: the title and conclusion may sound broader than the design supports.

What the authors should do: state clearly that the conclusions apply to two-point methods anchored at 100% body weight. The title, abstract, and conclusion should be adjusted, or the limitation should be stated more explicitly.

Response: We fully agree that, because all two-point methods in the present study used 100% BW as the lower anchor, our findings identify the best-performing two-point loading strategy among those anchored at 100% BW, rather than the globally optimal two-point loading strategy. We have therefore expanded the Limitations section to make this constraint explicit. Specifically, we have added the following paragraph to the Limitations subsection of the Discussion:

“A further limitation pertains to the loading design of the two-point methods. In the present study, all two-point methods were anchored at 100% BW as the lowest load, while only the heaviest load was varied from 120% to 300% BW. As a consequence of this design, the present findings identify the best-performing two-point loading combinations among those anchored at 100% BW, rather than the globally optimal two-point loading strategy across all possible anchor-load combinations. It remains unknown whether two-point methods using a lower anchor load (e.g., 60% or 80% BW), a higher anchor load, or load pairs not anchored at body weight would yield comparable, superior, or inferior agreement with the multiple-point reference method. Future studies should systematically examine how varying both the lower and upper anchor loads affects the agreement of two-point F–V profiling in horizontal dynamic leg press exercises.” (lines 353–363)

5. The resistance type is device-specific and its practical implications are underdeveloped

This is, in my view, a central issue. The study used a horizontal leg press with pneumatic artificial muscles and pneumatic semi-isotonic resistance. The authors note that this device “differs somewhat from other leg press machines” and that this may limit generalizability, but they move too quickly from that limitation to the suggestion that the two-point method should also be useful on other devices.

Why this matters: pneumatic resistance is not mechanically the same as a conventional plate-loaded or weight-stack leg press. A plate-based machine involves gravity and external mass inertia, whereas the current setup uses air-pressure-based semi-isotonic resistance. The manuscript itself states that this resistance prevented ballistic action. That means the movement mechanics, acceleration pattern, and possibly the force and velocity outputs may differ from those on conventional machines. Because the paper is identifying “optimal” load pairs, the type of resistance is not a minor technical detail. It is directly linked to whether the recommended load combinations can be transferred to other devices.

What the authors should do: substantially expand the Discussion on this point. They should explain why this pneumatic system was chosen, how it differs in practical terms from conventional plate-loaded machines, and why readers should or should not expect the same two-point load recommendations to apply elsewhere. The practical relevance section should be rewritten so that coaches and practitioners understand whether the recommendation is mainly for users of similar pneumatic systems, or whether broader transfer is actually supported.

Response: We are sincerely grateful to the Reviewer for raising this central methodological concern. As suggested, in the revised manuscript, we have clarified (i) why the pneumatic ddrobotec® device was selected, (ii) how it differs mechanically from conventional plate-loaded systems, and (iii) what readers can and cannot reasonably expect to transfer to other devices.

(i) Methodological rationale for selecting the pneumatic device: We have inserted a new sentence into the Limitations section explaining that this device was selected because of a fundamental methodological prerequisite of the present study itself: the validity of a two-point F–V profiling method can only be meaningfully evaluated on a measurement system that provides force and velocity outputs with high test–retest reliability. This is because measurement variability propagates more strongly into parameter estimates derived from only two experimental points than into those derived from a larger number of points [García-Ramos, 2023]. If the underlying force and velocity outputs were noisy, any observed disagreement between two-point and multiple-point methods could not be cleanly attributed to the loading design itself, because measurement error would confound the comparison. Our previous work has established that the ddrobotec® System ELITE mk4 provides leg press force, velocity, and power outputs with very high reliability [1], thereby satisfying this prerequisite and making the device a methodologically appropriate platform on which to evaluate alternative loading designs.

(ii) Mechanical differences from conventional plate-loaded systems: The Limitations section already cites Avrillon et al. [34] to indicate that the measured F–V relationship changes systematically with loading type (e.g., pneumatic vs. isoinertial), reflecting the differences in inertial mass and momentum carry-through between pneumatic semi-isotonic resistance and gravity-based plate-loaded or weight-stack systems. This passage has been retained, with the surrounding text revised to make the implications for cross-device transferability more explicit.

(iii) What does and does not transfer to other devices: We have revised the final clause of the relevant section in the Limitations to replace our previous overly broad assertion (“suggesting that the two-point method is also useful when leg press tasks are performed on other devices”) with a more precise distinction between two aspects of our findings:

The two-point methods using widely separated loads provide more accurate F–V parameter estimates than methods using closely spaced loads. This is expected to transfer across leg press devices on which the F–V relationship is quasi-linear. This expectation is grounded in two well-established findings: the F–V relationship in leg press tasks has been consistently shown to be quasi-linear across diverse loading modalities [6, 15, 35, 36], and the geometry of linear regression dictates that the slope of a linear relationship is estimated more stably as the distance between the two extreme experimental points increases [García-Ramos, 2023]. The principle is therefore grounded in the mathematics of linear regression rather than in device-specific resistance properties.

In contrast, the specific numerical load combinations identified here (100% paired with 240–300% BW) and the absolute magnitudes of F–V parameters may differ between loading modalities due to the systematic mechanical differences described in point (ii). We have therefore revised the text to state explicitly that these specific load combinations should be empirically confirmed on other systems before being adopted as device-specific recommendations, as follows:

“This device was selected in the present study because the validity of two-point F–V profiling could only be meaningfully evaluated on a measurement system providing force and velocity outputs with high test–retest reliability, since measurement variability propagates more strongly into estimates derived from only two experimental points than into those derived from a larger number of points [18].” (lines 365–369)

“However, based on previous studies [6,15,35,36] indicating that the F–V relationship for leg press tasks is quasi-linear across various load types, the principle that two-point methods using more distant loads provide more accurate F–V parameter estimates than methods using closer loads is expected to apply when leg press tasks are performed on other devices. This is grounded in the mathematics of linear regression rather than in device-specific properties [18]. However, the specific load combinations identified here may differ across devices and should be empirically confirmed on other systems.” (lines 372–378)

Together with the Conclusions section, which already restricts the practical recommendation to the tested device and population context, we believe that these revisions now (a) explicitly ground the cross-device generalizability claim in established theoretical evidence rather than in unsupported assertion, (b) clearly delineate which aspects of our findings transfer to other devices and which do not, and (c) provide readers with a principled basis for deciding how to apply our results to their own equipment contexts. We are grateful to the Reviewer for prompting this important strengthening of the Discussion.

6. Generalizability is limited and the conclusions should be narrowed

The study involved sixteen young men who were able to complete testing up to 300% body weight. The authors appropriately note that applicability to women and older adults remains unclear.

Why this matters: the final conclusion currently reads too broadly for a sample this narrow and a setup this specific.

What the authors should do: further restrict the conclusion to the tested population and device context. The practical message should not imply broad generalization to all users, all leg press systems, or all applied settings.

Response: We fully agree that the conclusions were too broadly framed for the specific sample and device context tested in this study. We have therefore substantially revised the Conclusions section to restrict our practical message to the tested population and device, and to explicitly limit claims of broader generalization. The revised Conclusions now read as follows.

“Therefore, the two-point method using widely separated loads can serve as a practical alternative to the multiple-point method for assessing leg extension capability, at least in young, relatively strong men tested on a horizontal dynamic leg press equipped with pneumatic artificial muscles. Generalization to other populations and leg press devices warrants further investigation.” (lines 391–395)

7. Reproducibility of data processing is not yet sufficient

The paper states that force and displacement were recorded at 200 Hz and that displacement was converted using system-specific pre-calibration data to estimate joint angles. However, the description remains too brief to permit full replication. It is not clear whether filtering was applied, whether part of the processing is proprietary, or how exactly the angle estimation procedure works.

Why this matters: this is a measurement paper, so reproducibility is especially important.

What the authors should do: expand the Methods section to describe the processing pipeline more completely. Please state whether filtering was used, how the angle estimation was derived, and whether raw or processed individual-level data are available in the supplement.

Response: As you suggested, we have expanded the Methods section to describe the processing pipeline in more detail. Furthermore, we have stated that, given the upper limit of this device’s sampling frequency (200 Hz), no digital filter was used to avoid over-smoothing, and we have clarified how the knee angle estimation was derived as follows. The individual-level data used in statistical analyses are available in the supplementary file “Original data.”

“Given the upper limit of this device’s sampling frequency (200 Hz), no digital filters were applied to these data to avoid over-smoothing.” (lines 136–138)

“Before the first measurement session, each participant was calibrated once using the manufacturer’s two-point procedure: with the lower back firmly against the backrest, the pedal positions corresponding to 5° and 90° of knee flexion were recorded. The device’s internal algorithm subsequently mapped pedal angle to anatomical knee angle from these two reference points across the full range of motion.” (lines 139–144)

Minor comments

The abstract is clear, but the final sentence is too broad. It should be limited to the tested horizontal pneumatic leg press context.

Response: In response to your feedback, we have revised the sentence.

“These findings indicate that, on a horizontal leg press with pneumatic artificial muscles, a two-point method based on sufficiently distinct loads can provide an accurate estimate of the F–V relationship and may be a practical alternative to comprehensive multi-load testing.” (lines 40–43)

The Introduction would benefit from a clearer explanation of why horizontal leg press is the preferred model for this question rather than another lower-limb task.

Response: We agree that the rationale for selecting the horizontal dynamic leg press as the testing model deserves a clearer presentation, and we would like to respectfully note that this rationale is already addressed in the first paragraph of the Introduction. Specifically, we identified two principal advantages of the horizontal dynamic leg press over other lower-limb tasks (e.g., vertical jumps):

1. Superior measurement reliability: Our previous work demonstrated that force, velocity, and power measurements during the horizontal dynamic leg press exhibit high test–retest reliability, with several variables showing higher reliability than corresponding measures obtained during vertical jumps.
2. Reduced injury risk: Unlike vertical jumping, the horizontal dynamic leg press does not involve a landing impact, allowing repeated maximal-effort trials (including under heavy loads) to be performed with substantially lower musculoskeletal risk. This is particularly relevant for F–V profiling, which inherently requires testing across a wide range of loads, including heavier loads.

These two features are precisely the properties required of a testing model used to validate two-point F–V profiling methods. To make this rationale more salient to the reader, we have revised the closing sentence of the first paragraph of the Introduction as follows:

“Taken together, the high measurement reliability and the absence of landing impact can make the horizontal dynamic leg press a particularly suitable model for evaluating F–V profiling methodology, in which a wide range of loads must be repeatedly tested.” (lines 54–56)

The Participants section should state how “men” was determined and whether sex was self-reported. Since only one sex was included, this needs clearer reporting.

Response: We recorded participants’ self-reported sex in this study. Therefore, we have added the following sentence to clarify the methods:

“All participants self-reported their sex as male.” (line 107)

Please justify the inclusion of only men more explicitly, or acknowledge it as a convenience sample.

Response: We recruited only men, who generally have greater lower-limb strength than women, because the study required data under heavier loads. Therefore, we have added the following sentence.

“Only men were recruited for the present study because the participants were required to perform the leg press exercise with heavier loads, and men generally have greater lower-limb strength than women.” (lines 104–107)

The familiarization-to-testing interval of 48–144 hours is quite wide. Please explain why this range was allowed.

Response: The difference in the interval between the finalization and testing sessions among participants was due to their availability as well as that of the investigators. Souza et al. showed acceptable reliability of countermovement jump height (ICC = 0.94, CV = 5.8%) across testing sessions separated by 2 to 7 days. Lindberg et al. also reported acceptable reliability of leg press force–velocity profile variables (ICC: 0.82–0.98, CV: 3.7–8.3%) across testing sessions separated by 1 week. Furthermore, short intervals (e.g., 24 hours) can lead to inadequate recovery from the familiarization session and inconsistent performance. Given these findings, the familiarization-to-testing interval of 48–144 hours is considered acceptable and appropriate to reduce fatigue and the potential for familiarization decay. We have added the references to our manuscript as follows.

“A familiarization session was conducted on the first day, and the main performance measurement was obtained on the second day, which was set 48–144 hours later depending on the availability and convenience of the participants and investigators, in accordance with previous studies [6,22].” (lines 89–92)

References

Souza AA, Bottaro M, Rocha VA, Lage V, Tufano JJ, Vieira A. Reliability and test-retest agreement of mechanical variables obtained during countermovement jump. Int J Exerc Sci. 2020;13: 6-17. doi:10.70252/XQXF8049

Lindberg K, Solberg P, Bjørnsen T, Helland C, Rønnestad B, Thorsen Frank M, et al. Force-velocity profiling in athletes: Reliability and agreement across methods. PLOS One. 2021;16: e0245791. doi: 10.1371/journal.pone.0245791

Rest intervals were also given as ranges rather than fixed durations. Please clarify how rest was standardized across participants and loads.

Response: We acknowledge that rest intervals were not standardized to a single fixed duration for each participant and external load condition. Instead, rest periods were managed within prescribed ranges (10–20 s for the first three external load levels and 20–40 s for the remaining higher load levels), with the next trial initiated after confirming each participant’s readiness through brief verbal communication (e.g., recovery of breathing and stable foot placement on the footplates). These prescribed rest interval ranges were identical to those used in our previous study, which employed the same horizontal dynamic leg press device. Additionally, we have added the references supporting the rest intervals to our manuscript as follows:

“The rest periods progressively increased with increasing load; for the first three load levels, the rest intervals ranged from 10 to 20 seconds, and for the remaining loads, from 20 to 40 seconds [1,5,6].” (lines 123–125)

The wording around force being summed across pedals but “average values for both legs” being used is not fully clear. Please clarify this calculation path.

Response: Thank you for pointing this out. We used the average values measured from both pedals for the velocity analysis, but not for the force analysis. To avoid any misunderstanding, we have revised the sentence in the Data analyses section as follows:

“Velocity values were computed as the average of the velocities measured from both pedals.” (lines 149–150)

The terminology around body weight, body mass, and external load should be made more consistent.

Response: We have made a clear distinction between these three terms. “Body mass” (kg) describes the participants’ anthropometric mass. “Body weight” (N) is used as the reference quantity for external load: the horizontal dynamic leg press device used in this study applied external loads (N) calibrated to each participant’s body weight, making “% body weight” a dimensionally appropriate descriptor of the applied resistance. “External load” refers to the resistance produced by the leg press device. We have verified that each term is used consistently with these definitions throughout the revised manuscript.

The paper should explain more clearly what practical size of bias would be acceptable for field use.

Response: We interpreted a mean percentage bias of less than 5% (good agreement) as acceptable bias for field use based on previous research. To clarify this interpretation, we have revised the manuscript as follows:

“The mean percent bias was interpreted a priori as poor (>10%), moderate (5–10%), or good (<5%), based on percent bias thresholds reported in previous research [27].” (lines 185–186)

“In fact, the bias when two-point methods based on distant loads (e.g., 100% and 180%, 100% and 300% BW) were compared to the multiple-point method was good (mean percent bias < 5%) for all F–V parameters.” (lines 212–215)

“The results indicate that only four two-point methods using different loads (100% and 240%, 100% and 260%, 100% and 280%, and 100% and 300% BW) provided nearly perfect correlations with the F–V relationship obtained from the multiple-point method and showed trivial to small differences and acceptable bias (<5%) for field use in all F–V parameters compared with those from the multiple-point method.” (lines 278–282)

Table 2 appears to include a duplicated row for the 100% and 120% condition in the text extract shown. Please check the final table carefully.

Response: Thank you for pointing this out. We have corrected Table 2.

Figure 1 caption is long and difficult to read. It could be simplified.

Response: In response to your comment, we have revised the caption of Figure 1 as follows.

“Figure 1. Typical leg press force–velocity (F–V) relationships of three individuals (A, B, C) showing different F–V profiles. The black solid line in each panel represents the regression line from the multiple-point method using all loads, with its formula inserted. The dotted lines represent 10 two-point methods, each combining 100% of body weight (BW) with a heavier load ranging from 120% to 300% of BW in 20% increments. Two-point method load pairs with 100% BW: 300% (black), 280% (gray), 260% (dark red), 240% (red), 220% (blue), 200% (light blue), 180% (green), 160% (light green), 140% (orange), 120% (purple).” (lines 163–169)

The manuscript says all relevant data are within the manuscript and supporting information. Please ensure the individual data used for F–V modeling are actually easy to locate in the supplement.

Response: We have ensured that the individual data used for F–V modeling are included in the supplementary file titled “Original data.”

The conclusion should avoid broad wording such as “useful in practice” unless the authors specify that this applies primarily to similar devices and populations.

Response: We agree that the original wording “useful … in practice” was overly broad relative to the scope of evidence provided by this single-device, single-population study, and that it was not fully consistent with the limitations we acknowledged in the Discussion. We have revised the final sentence of the Conclusions to explicitly restrict the practical claim to the device type (horizontal dynamic leg press with pneumatic artificial muscles) and the studied population (young, relatively strong men), and to flag that generalization beyond these conditions requires further investigation as follows:

“Therefore, the two-point method using widely separated loads can serve as a practical alternative to the multiple-point method for assessing leg extension capability, at least in young, relatively strong men tested on a horizontal dynamic leg press equipped with pneumatic artificial muscles. Generalization to other populations and leg press devices warrants further investigation.” (lines 391–395)

Recommendation to the editor

Major Revision

This is a useful and potentially publishable applied study, but it still needs substantial revision. The main concerns are not fatal flaws in the basic design. Rather, they relate to overstatement of validity, insufficient method-agreement framing, incomplete discussion of the resistance type, limited generalizability, and missing detail on reproducibility. The issue of pneumatic versus conventional leg press resistance is especially important because it affects both interpretation and practical transfer of the findings.

Response: We are sincerely grateful to the Reviewer for the thoughtful and constructive overall recommendation and for the encouraging assessment that the present study is “useful and potentially publishable.” We also fully accept the Reviewer's judgment that substantial revision was warranted, and we have undertaken a comprehensive revision that directly addresses each of the five overarching concerns raised in the recommendation. The specific revisions are detailed in our responses above. We hope these modifications effectively address the Reviewer’s concerns.
