# Supplementary material for: Optimal two-point loading strategy for accurate leg press force–velocity assessment
Source: PLoS One. 2026 Aug 25;21(8):e0356566. doi: 10.1371/journal.pone.0356566 (PMC13505900; doi:10.1371/journal.pone.0356566)
Supplement: Peer review history

# Peer review: Decision Letter 0

**DOI:** 10.1371/journal.pone.0356566.r001

**Authors:** Zixiang Gao

23 Apr 2026

Dear Dr. Inami,

Thank you for submitting your manuscript to PLOS ONE. After careful consideration, we feel that it has merit but does not fully meet PLOS ONE’s publication criteria as it currently stands. Therefore, we invite you to submit a revised version of the manuscript that addresses the points raised during the review process.

==============================

This manuscript addresses a relevant practical question and offers potentially useful findings; however, it requires major revision before it can be considered further. In particular, the claims regarding validity should be presented more cautiously, the agreement analysis should be strengthened, and the discussion should better address the biomechanical significance and limited generalizability associated with the pneumatic leg press system.

==============================

Please submit your revised manuscript by Jun 07 2026 11:59PM. If you will need more time than this to complete your revisions, please reply to this message or contact the journal office at plosone@plos.org. When you're ready to submit your revision, log on to https://www.editorialmanager.com/pone/ and select the 'Submissions Needing Revision' folder to locate your manuscript file.

- A letter that responds to each point raised by the academic editor and reviewer(s). You should upload this letter as a separate file labeled 'Response to Reviewers'.
- A marked-up copy of your manuscript that highlights changes made to the original version. You should upload this as a separate file labeled 'Revised Manuscript with Track Changes'.
- An unmarked version of your revised paper without tracked changes. You should upload this as a separate file labeled 'Manuscript'.

If you would like to make changes to your financial disclosure, please include your updated statement in your cover letter. Guidelines for resubmitting your figure files are available below the reviewer comments at the end of this letter.

If applicable, we recommend that you deposit your laboratory protocols in protocols.io to enhance the reproducibility of your results. Protocols.io assigns your protocol its own identifier (DOI) so that it can be cited independently in the future. For instructions see: https://journals.plos.org/plosone/s/submission-guidelines#loc-laboratory-protocols. Additionally, PLOS ONE offers an option for publishing peer-reviewed Lab Protocol articles, which describe protocols hosted on protocols.io. Read more information on sharing protocols at https://plos.org/protocols?utm_medium=editorial-email&utm_source=authorletters&utm_campaign=protocols.

As the corresponding author, your ORCID iD is verified in the submission system and will appear in the published article. PLOS supports the use of ORCID, and we encourage all coauthors to register for an ORCID iD and use it as well. Please encourage your coauthors to verify their ORCID iD within the submission system before final acceptance, as unverified ORCID iDs will not appear in the published article. *Only*  the individual author can complete the verification step; PLOS staff *cannot*  verify ORCID iDs on behalf of authors.

We look forward to receiving your revised manuscript.

Kind regards,

Zixiang Gao

Academic Editor

PLOS One

Journal Requirements:

When submitting your revision, we need you to address these additional requirements.

1. Please ensure that your manuscript meets PLOS ONE's style requirements, including those for file naming. The PLOS ONE style templates can be found at

https://journals.plos.org/plosone/s/file?id=wjVg/PLOSOne_formatting_sample_main_body.pdf and

https://journals.plos.org/plosone/s/file?id=ba62/PLOSOne_formatting_sample_title_authors_affiliations.pdf.

2. We note that there is identifying data in the Supporting Information file <Original data. >. Due to the inclusion of these potentially identifying data, we have removed this file from your file inventory. Prior to sharing human research participant data, authors should consult with an ethics committee to ensure data are shared in accordance with participant consent and all applicable local laws.

Data sharing should never compromise participant privacy. It is therefore not appropriate to publicly share personally identifiable data on human research participants. The following are examples of data that should not be shared:

-Name, initials, physical address

-Ages more specific than whole numbers

-Internet protocol (IP) address

-Specific dates (birth dates, death dates, examination dates, etc.)

-Contact information such as phone number or email address

-Location data

-ID numbers that seem specific (long numbers, include initials, titled “Hospital ID”) rather than random (small numbers in numerical order)

Data that are not directly identifying may also be inappropriate to share, as in combination they can become identifying. For example, data collected from a small group of participants, vulnerable populations, or private groups should not be shared if they involve indirect identifiers (such as sex, ethnicity, location, etc.) that may risk the identification of study

participants.

Additional guidance on preparing raw data for publication can be found in our Data Policy (https://journals.plos.org/plosone/s/data-availability#loc-human-research-participant-data-and-other-sensitive-data) and in the following article: http://www.bmj.com/content/340/bmj.c181.long.

Please remove or anonymize all personal information (<specific identifying information in file to be removed>), ensure that the data shared are in accordance with participant consent, and re-upload a fully anonymized data set. Please note that spreadsheet columns with personal information must be removed and not hidden as all hidden columns will appear in the published file.

3. If the reviewer comments include a recommendation to cite specific previously published works, please review and evaluate these publications to determine whether they are relevant and should be cited. There is no requirement to cite these works unless the editor has indicated otherwise.

[Note: HTML markup is below. Please do not edit.]

Reviewers' comments:

Reviewer's Responses to Questions

**Comments to the Author**

1. Is the manuscript technically sound, and do the data support the conclusions?

Reviewer #1: Yes

Reviewer #2: Partly

Reviewer #3: Partly

**********

2. Has the statistical analysis been performed appropriately and rigorously?

Reviewer #1: Yes

Reviewer #2: Yes

Reviewer #3: N/A

**********

3. Have the authors made all data underlying the findings in their manuscript fully available?

The PLOS Data policy

Reviewer #1: Yes

Reviewer #2: Yes

Reviewer #3: Yes

**********

4. Is the manuscript presented in an intelligible fashion and written in standard English?

Reviewer #1: Yes

Reviewer #2: Yes

Reviewer #3: Yes

**********

Reviewer #1: 1. The current analysis relies on Pearson's r, paired t-tests, and Cohen's d. While useful, Pearson's r evaluates linear correlation rather than absolute agreement between two methods.

2. The authors should clarify whether a sample size of n=16 provides sufficient statistical power to detect meaningful differences in the validity assessments between the various two-point models.

3. The authors acknowledge the use of a pneumatic system as a limitation regarding generalizability. It would significantly strengthen the discussion to briefly explain why this matters from a biomechanical perspective.

4. There is a duplication error in Table 2.

Reviewer #2: General comment

I congratulate the authors on their efforts in conducting this study, which aimed to examine the concurrent validity of the F–V relationship during a horizontal leg press exercise by comparing two-point methods with a multiple-point method. The results indicated that the F–V relationship obtained from the four two-point methods with wider load differences (100% and 240%, 100% and 260%, 100% and 280%, and 100% and 300% BW) was nearly perfectly correlated with that obtained by the multiple-point method (r = 0.906-0.993). The concurrent validity of the F–V relationship parameters obtained from the two-point methods tended to decrease when using loads that were closer together (e.g., 100% and 120% BW) than loads with a larger difference (e.g., 100% and 240% BW). Although the article presents important content for journal readers, indicating that, on a horizontal leg press, a two-point method based on sufficiently distinct loads can provide an accurate estimate of the F–V relationship and may be a practical alternative to comprehensive multiple-load testing, it has some clear limitations: a small sample size and the use of a pneumatic leg press machine limit the generalizability of the findings. Furthermore, the study's practical applications should be reinforced. Therefore, the manuscript needs further clarification.

Below, I provide some suggestions.

Introduction

Page 4, LL44: “Leg extensions are performed during various athletic activities...”

I suggest that authors provide examples of these activities.

Page 4, LL46: “We [1] recently reported high reliability…”

I recommend that authors write the beginning of the sentence more formally. For example, you might consider beginning with: "Our research team recently reported."

Materials and Methods

Participants

Page 6, LL90-91: “The sample size was calculated using the G*Power software…”

The study included a sample size calculation, thereby providing added value.

Page 6, LL92-94: “Based on an expected correlation coefficient of 0.7, a significance level of 0.05, and a statistical power of the required sample size was calculated to be 16 participants.”

The authors should support the sample size calculation with appropriate references.

Testing procedures

Page 7, LL113-115: “The rest periods progressively increased with increasing load; for the first three load levels, the rest intervals ranged from 10 to 20 seconds, and for the remaining loads, from 20 to 40 seconds.”

What evidence do the authors use to support these rest periods?

Page 7, LL116-118: “Pneumatic semi-isotonic resistance prevented maximal effort from producing ballistic action, and the entire push-off was performed with maximal intentional velocity.”

Please, add references.

Data analyses

Page 8, LL137: Have multiple regression analyses been considered for this analysis?

Statistical analyses

Page 10, LL137: Measurement reliability.

I recommend that the authors separate the reliability analysis into two parts for improved accuracy: relative reliability (ICC) and absolute reliability (CV and SEM).

Results

The results subsection is well-articulated and accurately presented.

Discussion

Page 15, LL225-227: “This study tested the hypothesis that the concurrent validity of the F–V relationship parameters obtained from the two-point method is high when different loads are used, whereas this validity progressively decreases when closer loads are used.”

I recommend that the authors revise the paragraph to enhance its clarity and overall comprehension of the study's theme.

Page 16, LL233-234: “These results support our hypotheses.”

The sentence appears isolated; the authors should integrate the idea into another sentence within the paragraph.

Page 16, LL235-236: “Reliable F–V profiling under multiple load conditions requires that the force and velocity of each input data point be highly reliable.”

I recommend that authors include reference values to clarify which metrics can be deemed highly reliable.

Page 16, LL246-248: “These findings suggest that data points obtained under very high load conditions, where the mean velocity falls below approximately 0.5 m/s, should be excluded to enhance the reliability of leg press F–V profiling.”

The authors should explore in greater depth the factors that contribute to the diminished reliability of velocity-related indicators under very high load conditions.

Conclusion

Page 19, LL299-300: “Specifically, applying relatively distant loads in the two-point method enables the acquisition of more valid F–V parameters.”

I suggest that the authors include examples of the load values.

Reviewer #3: Overall assessment

This manuscript addresses a relevant applied question in sports science: whether a simpler two-point loading method can provide a valid estimate of the force–velocity (F–V) profile during horizontal leg press exercise, compared with a more time-consuming multiple-point approach. The study is focused, the design is clear, and the main practical message is potentially useful. The results suggest that two-point methods using more widely separated loads perform better than methods using closer loads, and that some two-point combinations may approximate the multi-point method reasonably well.

The manuscript has merit and could become publishable after revision. However, several points need stronger treatment before acceptance. The main issues concern the framing of validity, the agreement analysis, the limited generalizability of the findings, and especially the device-specific nature of the resistance used. The practical implications of using a pneumatic semi-isotonic leg press rather than a conventional plate-loaded machine are not explained well enough.

Summary of the manuscript

The study compares ten two-point approaches for estimating leg press F–V parameters with a multiple-point method based on eleven loads from 100% to 300% body weight. Sixteen young men performed maximal horizontal leg press actions on a device equipped with pneumatic artificial muscles. The authors calculated F0, V0, Pmax, and slope, and compared the two-point estimates with the multiple-point estimates using paired tests, effect sizes, correlations, and bias. The main finding is that two-point methods using larger load separation, especially 100% paired with 240–300% body weight, showed the best agreement with the multiple-point method.

Strengths

The topic is practically important for both researchers and practitioners interested in reducing testing burden.

The study asks a clear question and tests multiple load pairings rather than only one candidate two-point approach.

The manuscript reports reliability metrics across loads, which helps readers judge measurement quality.

The main result is easy to interpret and likely useful in practice within the tested context.

The Discussion acknowledges some limitations, including population specificity and device dependence, although these parts need expansion.

Major comments

1. The reference method is not a true gold standard, so the validity claim should be framed more carefully

The manuscript presents the analysis as a test of concurrent validity, but the multiple-point method is not an independent criterion method. It is another model-based estimate derived from the same session and the same measurement system. This makes the study a comparison against a broader-load reference approach rather than a validation against a true external standard.

Why this matters: the current wording risks overstating what has been shown.

What the authors should do: revise the wording throughout the manuscript to make clear that the study evaluates agreement or concurrent validity relative to a multi-point reference method, not criterion validity against an external gold standard. This is especially important in the Abstract, Discussion, and Conclusions.

2. Correlation is not sufficient to support interchangeability between methods

The statistical approach includes paired t-tests, effect sizes, Pearson correlations, and bias. This is useful, but correlation alone does not establish that two methods can be used interchangeably. A strong correlation can still coexist with practically important disagreement.

Why this matters: the paper’s practical claim is that a two-point method may replace a multi-point test. That requires a stronger agreement framework.

What the authors should do: add agreement-focused analysis for the preferred two-point methods, ideally Bland–Altman plots or a similar method-comparison approach. If the authors do not wish to add such analyses, the practical conclusions should be made more cautious.

3. The sample size justification does not fully match the main study aim

The sample size calculation appears to be based on an expected correlation coefficient and is described as being performed to assess reliability. However, the main aim of this paper is comparison between two-point and multiple-point methods.

Why this matters: the sample size rationale should align with the primary study objective.

What the authors should do: clarify whether the current sample size calculation was intended for reliability, method comparison, or both. If the study was powered only for a reliability-type analysis, this should be stated as a limitation.

4. The fixed use of 100% body weight as the lower load narrows the meaning of “optimal”

All two-point methods were anchored at 100% body weight, with only the heavier load varying. Therefore, the study does not identify the globally optimal two-point loading strategy. It identifies the best-performing strategy among methods anchored at 100% body weight.

Why this matters: the title and conclusion may sound broader than the design supports.

What the authors should do: state clearly that the conclusions apply to two-point methods anchored at 100% body weight. The title, abstract, and conclusion should be adjusted, or the limitation should be stated more explicitly.

5. The resistance type is device-specific and its practical implications are underdeveloped

This is, in my view, a central issue. The study used a horizontal leg press with pneumatic artificial muscles and pneumatic semi-isotonic resistance. The authors note that this device “differs somewhat from other leg press machines” and that this may limit generalizability, but they move too quickly from that limitation to the suggestion that the two-point method should also be useful on other devices.

Why this matters: pneumatic resistance is not mechanically the same as a conventional plate-loaded or weight-stack leg press. A plate-based machine involves gravity and external mass inertia, whereas the current setup uses air-pressure-based semi-isotonic resistance. The manuscript itself states that this resistance prevented ballistic action. That means the movement mechanics, acceleration pattern, and possibly the force and velocity outputs may differ from those on conventional machines. Because the paper is identifying “optimal” load pairs, the type of resistance is not a minor technical detail. It is directly linked to whether the recommended load combinations can be transferred to other devices.

What the authors should do: substantially expand the Discussion on this point. They should explain why this pneumatic system was chosen, how it differs in practical terms from conventional plate-loaded machines, and why readers should or should not expect the same two-point load recommendations to apply elsewhere. The practical relevance section should be rewritten so that coaches and practitioners understand whether the recommendation is mainly for users of similar pneumatic systems, or whether broader transfer is actually supported.

6. Generalizability is limited and the conclusions should be narrowed

The study involved sixteen young men who were able to complete testing up to 300% body weight. The authors appropriately note that applicability to women and older adults remains unclear.

Why this matters: the final conclusion currently reads too broadly for a sample this narrow and a setup this specific.

What the authors should do: further restrict the conclusion to the tested population and device context. The practical message should not imply broad generalization to all users, all leg press systems, or all applied settings.

7. Reproducibility of data processing is not yet sufficient

The paper states that force and displacement were recorded at 200 Hz and that displacement was converted using system-specific pre-calibration data to estimate joint angles. However, the description remains too brief to permit full replication. It is not clear whether filtering was applied, whether part of the processing is proprietary, or how exactly the angle estimation procedure works.

Why this matters: this is a measurement paper, so reproducibility is especially important.

What the authors should do: expand the Methods section to describe the processing pipeline more completely. Please state whether filtering was used, how the angle estimation was derived, and whether raw or processed individual-level data are available in the supplement.

Minor comments

The abstract is clear, but the final sentence is too broad. It should be limited to the tested horizontal pneumatic leg press context.

The Introduction would benefit from a clearer explanation of why horizontal leg press is the preferred model for this question rather than another lower-limb task.

The Participants section should state how “men” was determined and whether sex was self-reported. Since only one sex was included, this needs clearer reporting.

Please justify the inclusion of only men more explicitly, or acknowledge it as a convenience sample.

The familiarization-to-testing interval of 48–144 hours is quite wide. Please explain why this range was allowed.

Rest intervals were also given as ranges rather than fixed durations. Please clarify how rest was standardized across participants and loads.

The wording around force being summed across pedals but “average values for both legs” being used is not fully clear. Please clarify this calculation path.

The terminology around body weight, body mass, and external load should be made more consistent.

The paper should explain more clearly what practical size of bias would be acceptable for field use.

Table 2 appears to include a duplicated row for the 100% and 120% condition in the text extract shown. Please check the final table carefully.

Figure 1 caption is long and difficult to read. It could be simplified.

The manuscript says all relevant data are within the manuscript and supporting information. Please ensure the individual data used for F–V modeling are actually easy to locate in the supplement.

The conclusion should avoid broad wording such as “useful in practice” unless the authors specify that this applies primarily to similar devices and populations.

Recommendation to the editor

Major Revision

This is a useful and potentially publishable applied study, but it still needs substantial revision. The main concerns are not fatal flaws in the basic design. Rather, they relate to overstatement of validity, insufficient method-agreement framing, incomplete discussion of the resistance type, limited generalizability, and missing detail on reproducibility. The issue of pneumatic versus conventional leg press resistance is especially important because it affects both interpretation and practical transfer of the findings.

**********

what does this mean?). If published, this will include your full peer review and any attached files.

If you choose “no”, your identity will remain anonymous but your review may still be made public.

**Do you want your identity to be public for this peer review?**  For information about this choice, including consent withdrawal, please see our Privacy Policy

Reviewer #1: No

Reviewer #2: No

Reviewer #3: **Yes:**  Dragan Mirkov

**********

[NOTE: If reviewer comments were submitted as an attachment file, they will be attached to this email and accessible via the submission site. Please log into your account, locate the manuscript record, and check for the action link "View Attachments". If this link does not appear, there are no attachment files.]

To ensure your figures meet our technical requirements, please review our figure guidelines: https://journals.plos.org/plosone/s/figures

You may also use PLOS’s free figure tool, NAAS, to help you prepare publication quality figures: https://journals.plos.org/plosone/s/figures#loc-tools-for-figure-preparation.

NAAS will assess whether your figures meet our technical requirements by comparing each figure against our figure specifications.

# Author comment: Author response to Decision Letter 1

**DOI:** 10.1371/journal.pone.0356566.r002

2 Jun 2026

Thank you for the opportunity to resubmit our manuscript entitled, “Optimal two-point loading strategy for accurate leg press force–velocity assessment.” We have carefully reviewed the reviewers’ comments and revised the manuscript accordingly. All necessary changes have been made, and detailed responses to each comment are provided below. We believe our responses are adequate, the revised manuscript (PONE-D-25-63455-R1) has been improved, and meets the criteria for publication in the journal.

Reviewer #1

1. The current analysis relies on Pearson’s r, paired t-tests, and Cohen’s d. While useful, Pearson’s r evaluates linear correlation rather than absolute agreement between two methods.

Response: We fully agree with your comment. In response to your feedback, we have added Bland–Altman plots (Figures 2–5) showing that the two-point method using more distant loads provides better agreement with the multiple-point method. The following sentences have been added to the manuscript:

“Bland–Altman plots were generated, providing a comprehensive representation of the agreement between the two- and multiple-point methods.” (lines 191–192)

“Bland–Altman plots (Figures 2–5) demonstrated that the two-point method using more distant loads showed better agreement with the multiple-point method, whereas the two-point method using closer loads showed poorer agreement.” (lines 232–235)

2. The authors should clarify whether a sample size of n=16 provides sufficient statistical power to detect meaningful differences in the validity assessments between the various two-point models.

Response: The sample size of 16 was determined a priori using G*Power (version 3.1.9.6) with the following parameters: an expected correlation coefficient of r = 0.7 (based on García-Ramos et al. [20]), a significance level of α = 0.05, and a statistical power (1 − β) of 0.95. As clarified in the revised “Participants” section (lines 93–97), this calculation was specifically designed to detect a significant correlation between the F–V relationship parameters obtained from the two-point and multiple-point methods, which was the main aim of this study. The chosen power level (0.95) is more stringent than the conventional 0.80, providing a substantial safeguard against Type II error in the validity assessments. Furthermore, the resulting sample size is comparable to those used in previous validation studies of the two-point method for lower-limb tasks (e.g., García-Ramos et al. [20]), further supporting its adequacy for the present validity analyses. In addition, we have noted in the Conclusion section that our sample represents a specific population (young, relatively strong men tested on a single device), which may limit the generalizability of our findings (lines 388–393).

3. The authors acknowledge the use of a pneumatic system as a limitation regarding generalizability. It would significantly strengthen the discussion to briefly explain why this matters from a biomechanical perspective.

Response: Thank you very much for the valuable suggestion. Previous research (Avrillon et al., 2017) on the ballistic bench press has reported that the measured force–velocity (F–V) relationship varies depending on the loading modality. Specifically, pneumatic resistance mitigates inertial influences compared to isoinertial resistance, thereby limiting the magnitude of the initial force required to displace the barbell. These findings suggest that the loading modality affects the F–V relationship, highlighting it as a critical factor to be considered in its measurement. Accordingly, while we have acknowledged the use of a pneumatic leg press device with artificial muscles as a limitation of the present study, it is important to note that the F–V relationship in leg press tasks has been consistently shown to follow a linear trend across various loading patterns. Based on this rationale, we have addressed the generalizability of our findings to other leg press devices in the revised Discussion. Furthermore, we have revised the manuscript to more clearly state our intentions behind addressing the loading modality as follows:

“This difference in loading type may limit the generalizability of the present study’s findings when other leg press devices are used because the measured F–V relationship changes depending on the loading type (e.g., pneumatic vs. isoinertial) [34].” (lines 369–371)

4. There is a duplication error in Table 2.

Response: Thank you very much for pointing that out. We have corrected the duplication error in Table 2.

Reviewer #2

General comment

I congratulate the authors on their efforts in conducting this study, which aimed to examine the concurrent validity of the F–V relationship during a horizontal leg press exercise by comparing two-point methods with a multiple-point method. The results indicated that the F–V relationship obtained from the four two-point methods with wider load differences (100% and 240%, 100% and 260%, 100% and 280%, and 100% and 300% BW) was nearly perfectly correlated with that obtained by the multiple-point method (r = 0.906-0.993). The concurrent validity of the F–V relationship parameters obtained from the two-point methods tended to decrease when using loads that were closer together (e.g., 100% and 120% BW) than loads with a larger difference (e.g., 100% and 240% BW). Although the article presents important content for journal readers, indicating that, on a horizontal leg press, a two-point method based on sufficiently distinct loads can provide an accurate estimate of the F–V relationship and may be a practical alternative to comprehensive multiple-load testing, it has some clear limitations: a small sample size and the use of a pneumatic leg press machine limit the generalizability of the findings. Furthermore, the study’s practical applications should be reinforced. Therefore, the manuscript needs further clarification.

Below, I provide some suggestions.

Response: We sincerely thank the reviewer for their careful evaluation and for noting that our study provides important content for journal readers. We particularly appreciate the accurate summary of our key finding, namely that the two-point methods using more widely separated loads (100% and 240–300% BW) yielded F–V parameters that were nearly perfectly correlated with those obtained from the multiple-point method (r = 0.906–0.993).

We also acknowledge the three concerns raised in the overall assessment: (i) the sample size, (ii) the use of a pneumatic leg press machine, and (iii) the need to reinforce the practical applications. We address these concerns as follows and address each specific suggestion in the point-by-point replies below.

(i) The sample size of 16 was determined a priori using G*Power (expected r = 0.7 based on García-Ramos et al. [20], α = 0.05, power = 0.95), as described in the Participants section. This sample size is comparable to those reported in previous validation studies of the two-point method for lower-limb tasks (e.g., García-Ramos et al. [20]). In addition, we have noted in the Conclusion section that our sample represents a specific population, which may limit the generalizability of our findings (lines 391–395).

(ii) We agree that the use of a pneumatic horizontal dynamic leg press device (ddrobotec® System ELITE) is a device-specific feature of our study. As noted in the Discussion (lines 372–378), however, the quasi-linear nature of the F–V relationship in leg press tasks has been consistently reported across different loading modalities, which provides a theoretical basis for expecting that the two-point method can also be applied to other leg press devices.

(iii) We fully agree that the practical applications need to be addressed better. In the revised manuscript, we have expanded this section to include: (a) a concrete recommendation of optimal load pairs (100% combined with 240–300% BW) supported by our concurrent validity results; and (b) a quantification of the time- and fatigue-saving advantage (4 trials versus 22 trials in the present study) that the two-point method offers in field and clinical settings.

“Specifically, using relatively distant loads (100% and 240%, 100% and 260%, 100% and 280%, and 100% and 300% BW) in the two-point method enables the acquisition of more valid F–V parameters and provides a time-efficient, fatigue-reducing alternative (e.g., 4 vs. 22 trials in the present study).” (lines 388–395)

Introduction

Page 4, LL44: “Leg extensions are performed during various athletic activities...”

I suggest that authors provide examples of these activities.

Response: As you suggested, we have revised the sentence as follows:

“Leg extensions are performed during various athletic activities, such as vertical jumps or sprint running [1,2], and horizontal dynamic leg press exercises are widely used to train and evaluate leg extension performance [1,3–8].” (lines 45–47)

Page 4, LL46: “We [1] recently reported high reliability…”

I recommend that authors write the beginning of the sentence more formally. For example, you might consider beginning with: “Our research team recently reported.”

Response: As you suggested, we have revised the sentence as follows:

“Our research team recently reported high reliability in measuring force, velocity, and power during the horizontal dynamic leg press exercise using a device equipped with pneumatic artificial muscles (ddrobotec® System ELITE mk4, Dynamic Devices, Zurich, Switzerland), with some variables showing higher reliability than those measured in vertical jumps [1].” (lines 47–51)

Materials and Methods

Participants

Page 6, LL90-91: “The sample size was calculated using the G*Power software…”

The study included a sample size calculation, thereby providing added value.

Response: Thank you for your comments.

Page 6, LL92-94: “Based on an expected correlation coefficient of 0.7, a significance level of 0.05, and a statistical power of the required sample size was calculated to be 16 participants.”

The authors should support the sample size calculation with appropriate references.

Response: As you suggested, we have added the appropriate reference supporting the sample size calculation as follows:

“Based on an expected correlation coefficient of 0.7 [20], a significance level of 0.05, and a statistical power of 0.95, the required sample size was calculated to be 16 participants.” (lines 100–101)

Testing procedures

Page 7, LL113-115: “The rest periods progressively increased with increasing load; for the first three load levels, the rest intervals ranged from 10 to 20 seconds, and for the remaining loads, from 20 to 40 seconds.”

What evidence do the authors use to support these rest periods?

Response: In response to your comment, we have added previous studies that support the rest periods used in our study when using a horizontal leg press machine.

“The rest periods progressively increased with increasing load; for the first three load levels, the rest intervals ranged from 10 to 20 seconds, and for the remaining loads, from 20 to 40 seconds [1,5,6].” (lines 123–125)

Page 7, LL116-118: “Pneumatic semi-isotonic resistance prevented maximal effort from producing ballistic action, and the entire push-off was performed with maximal intentional velocity.”

Please, add references.

Response: As you suggested, we have added some references.

“Pneumatic semi-isotonic resistance prevented maximal effort from producing ballistic action, and the entire push-off was performed with maximal intentional velocity [1,5,6].” (lines 126–128)

Data analyses

Page 8, LL137: Have multiple regression analyses been considered for this analysis?

Response: This regression analysis was conducted to model the force–velocity relationship during the leg press exercise. It is known that the force–velocity relationship in multi-joint movements such as the leg press follows a quasi-linear pattern. Therefore, rather than using multiple regression analysis, we conducted a least-squares linear regression, as recommended in previous studies [6,20]. Thus, we believe that this analysis was appropriate.

Statistical analyses

Page 10, LL137: Measurement reliability.

I recommend that the authors separate the reliability analysis into two parts for improved accuracy: relative reliability (ICC) and absolute reliability (CV and SEM).

Response: In response to your feedback, we have revised the sentence as follows:

“The intraclass correlation coefficient (ICC; two-way mixed effects, absolute agreement, single measurement) [24] was calculated to analyze relative reliability, and the coefficient of variation (CV) and standard error of measurement (SEM) [25] were calculated to analyze absolute reliability.” (lines 175–178)

Results

The results subsection is well-articulated and accurately presented.

Response: Thank you very much.

Discussion

Page 15, LL225-227: “This study tested the hypothesis that the concurrent validity of the F–V relationship parameters obtained from the two-point method is high when different loads are used, whereas this validity progressively decreases when closer loads are used.”

I recommend that the authors revise the paragraph to enhance its clarity and overall comprehension of the study’s theme.

Response: As you recommended, we have revised the sentence to improve the clarity and highlight findings related to the main topic of the study in the last sentence of the paragraph.

“These results support our hypotheses and indicate that the two-point method using distant loads yields leg press F–V relationship parameters that are well matched to those from the multiple-point method.” (lines 285–287)

Page 16, LL233-234: “These results support our hypotheses.”

The sentence appears isolated; the authors should integrate the idea into another sentence within the paragraph.

Response: As you pointed out, that sentence is too short and isolated. However, combining it with other sentences risks making the sentence too long or causing it to deviate from the original point. Therefore, we have expanded the sentence as mentioned above (lines 285–287).

Page 16, LL235-236: “Reliable F–V profiling under multiple load conditions requires that the force and velocity of each input data point be highly reliable.”

I recommend that authors include reference values to clarify which metrics can be deemed highly reliable.

Response: As you suggested, we have included reference values in the sentence.

“Reliable F–V profiling under multiple load conditions requires that the force and velocity of each input data point be highly reliable (ICC > 0.75, CV < 10%) [20,26].” (lines 288–289)

Page 16, LL246-248: “These findings suggest that data points obtained under very high load conditions, where the mean velocity falls below approximately 0.5 m/s, should be excluded to enhance the reliability of leg press F–V profiling.”

The authors should explore in greater depth the factors that contribute to the diminished reliability of velocity-related indicators under very high load conditions.

Response: We agree that the original Discussion noted the empirical pattern but did not adequately explore its underlying mechanism. We have added the following explanations to the Discussion proposing that the velocity-specific reduction in reliability observed at 300% BW reflects increased sensitivity of the velocity calculations to between-trial variability in movement timing—particularly the timing of force initiation, left–right leg synchronization, and consistency of completion time—when participants are operating close to their maximal force capacity. We support this argument with reference to a recent systematic review (Bakhshinejad et al., 2025, Sports Med Open) that synthesized 22 biomechanical studies and concluded that joint coordination and segmental variability consistently increase with exercise intensity in experienced lifters. We also explicitly acknowledge that those previous findings were obtained with free-weight exercises having greater kinematic degrees of freedom than the present horizontal leg press device and clarify that between-trial variability persists even with the device’s mechanical standardization. The added paragraph also explicitly contrasts this velocity-specific phenomenon with th

Attachment Submitted filename: Response_to_Reviewers.docx

# Peer review: Decision Letter 1

**DOI:** 10.1371/journal.pone.0356566.r003

**Authors:** Zixiang Gao

5 Aug 2026

<p>Optimal two-point loading strategy for accurate leg press force–velocity assessment

PONE-D-25-63455R1

Dear Dr. Inami,

We’re pleased to inform you that your manuscript has been judged scientifically suitable for publication and will be formally accepted for publication once it meets all outstanding technical requirements.

Within one week, you’ll receive an e-mail detailing the required amendments. When these have been addressed, you’ll receive a formal acceptance letter and your manuscript will be scheduled for publication.

An invoice will be generated when your article is formally accepted. Please note, if your institution has a publishing partnership with PLOS and your article meets the relevant criteria, all or part of your publication costs will be covered. Please make sure your user information is up-to-date by logging into Editorial Manager at Editorial Manager® and clicking the ‘Update My Information' link at the top of the page. For questions related to billing, please contact billing support.

If your institution or institutions have a press office, please notify them about your upcoming paper to help maximize its impact. If they’ll be preparing press materials, please inform our press team as soon as possible -- no later than 48 hours after receiving the formal acceptance. Your manuscript will remain under strict press embargo until 2 pm Eastern Time on the date of publication. For more information, please contact onepress@plos.org.

Kind regards,

Zixiang Gao

Academic Editor

PLOS One

Additional Editor Comments (optional):

Reviewers' comments:

Reviewer's Responses to Questions

**Comments to the Author**

Reviewer #2: All comments have been addressed

**********

2. Is the manuscript technically sound, and do the data support the conclusions?

Reviewer #2: Yes

**********

3. Has the statistical analysis been performed appropriately and rigorously?

Reviewer #2: Yes

**********

4. Have the authors made all data underlying the findings in their manuscript fully available?

The PLOS Data policy

Reviewer #2: Yes

**********

5. Is the manuscript presented in an intelligible fashion and written in standard English?

Reviewer #2: Yes

**********

Reviewer #2: The authors have diligently addressed the recommendations provided, resulting in a substantial enhancement of the study's quality. I believe the study is now ready for publication. All the best

**********

what does this mean?). If published, this will include your full peer review and any attached files.

If you choose “no”, your identity will remain anonymous but your review may still be made public.

**Do you want your identity to be public for this peer review?**  For information about this choice, including consent withdrawal, please see our Privacy Policy

Reviewer #2: No

**********

# Editor report: Acceptance letter

**DOI:** 10.1371/journal.pone.0356566.r004

**Authors:** Zixiang Gao

PONE-D-25-63455R1

PLOS One

Dear Dr. Inami,

I'm pleased to inform you that your manuscript has been deemed suitable for publication in PLOS One. Congratulations! Your manuscript is now being handed over to our production team.

At this stage, our production department will prepare your paper for publication. This includes ensuring the following:

* All references, tables, and figures are properly cited

* All relevant supporting information is included in the manuscript submission,

* There are no issues that prevent the paper from being properly typeset

You will receive further instructions from the production team, including instructions on how to review your proof when it is ready. Please keep in mind that we are working through a large volume of accepted articles, so please give us a few days to review your paper and let you know the next and final steps.

Lastly, if your institution or institutions have a press office, please let them know about your upcoming paper now to help maximize its impact. If they'll be preparing press materials, please inform our press team within the next 48 hours. Your manuscript will remain under strict press embargo until 2 pm Eastern Time on the date of publication. For more information, please contact onepress@plos.org.

You will receive an invoice from PLOS for your publication fee after your manuscript has reached the completed accept phase. If you receive an email requesting payment before acceptance or for any other service, this may be a phishing scheme. Learn how to identify phishing emails and protect your accounts at https://explore.plos.org/phishing.

If we can help with anything else, please email us at customercare@plos.org.

Thank you for submitting your work to PLOS One and supporting open access.

Kind regards,

PLOS ONE Editorial Office Staff

on behalf of

Dr. Zixiang Gao

Academic Editor

PLOS One
